# Influence of web openings on the cyclic response of RC coupling beams

**DOI:** 10.1038/s41598-026-42360-y

**Published:** 2026-03-26

**Authors:** Osman M. O. Ramadan, Ahmed Elghool, Nasser Elshafey, Mohamed S. Elbayomy

**Affiliations:** 1 Department of Structural Engineering, Faculty of Engineering, Cairo University, Giza, Egypt; 2 Department of Structural Engineering, Faculty of Engineering, City University of Cairo, Badr City, Egypt

**Keywords:** Engineering, Materials science, Physics

## Abstract

The use of appropriate reinforcement detailing in reinforced concrete coupling beams is crucial, particularly for those with a short span-to-depth ratio (ln/h < 2). These beams must be designed to exhibit ductile, inelastic behavior, thereby maintaining the stability of the coupled shear wall system. In practice, openings are often required in coupling beams to accommodate utility pipes for plumbing, electrical, and other building services. However, these openings can significantly alter the structural behavior and must be accounted for in the design. Since experimental investigation of various parameters is often expensive and time-consuming, this study employs computational modeling. A set of twelve reinforced concrete coupling beams, with and without openings, were analyzed using the non-linear finite element program ABAQUS to investigate their cyclic behavior. The beams featured different reinforcement details: conventional, diagonal confinement, and two configurations of rhombic details. All beams had identical dimensions of 200 mm in width and 400 mm in both depth and length (ln/h = 1). The primary variables were the presence, location, and reinforcement scheme around the openings. The results indicate that the solid coupling beam with diagonal reinforcement exhibited overall behavior superior to those with conventional or rhombic details. The introduction of an opening with relative dimensions of (lo/l = 0.125 and ho/t = 0.25) had a significant detrimental effect, especially when located at the mid-span. The behavior of beams with diagonal confinement was less affected by the presence of an opening than the other details, regardless of its location. Providing an opening 37.5 mm from the beam end reduced the shear capacity and maximum chord rotation by (10.2%, 30.4%), (5.5%, 15.6%), and (12.78%, 55.2%) for the conventional, diagonal confinement, and rhombic configuration 1 details, respectively, compared to their solid counterparts. Shifting the opening to the mid-span increased these reductions to (31.6%, 58.8%), (14.5%, 48.8%), (26.4%, 65.6%), and (19.2%, 50.2%) for the conventional, diagonal confinement, rhombic configuration 1, and rhombic configuration 2 details, respectively.

## Introduction

Reinforced concrete shear walls are a common lateral load-resisting system in high-rise buildings located in seismic zones. Cores requiring access for elevators and other utilities often feature aligned openings, which effectively convert a single wall into a coupled shear wall system. The spandrel, or coupling/link beam, connects these adjacent walls and acts as a structural “fuse”. Effective reinforcement detailing in RC elements is necessary to provide critical zones with the adequate ductility desired for global seismic performance^1^. Consequently, special reinforcement details are recommended for coupling beams (CBs) to ensure they can dissipate energy and undergo large inelastic rotations.

**Fig. 1:**
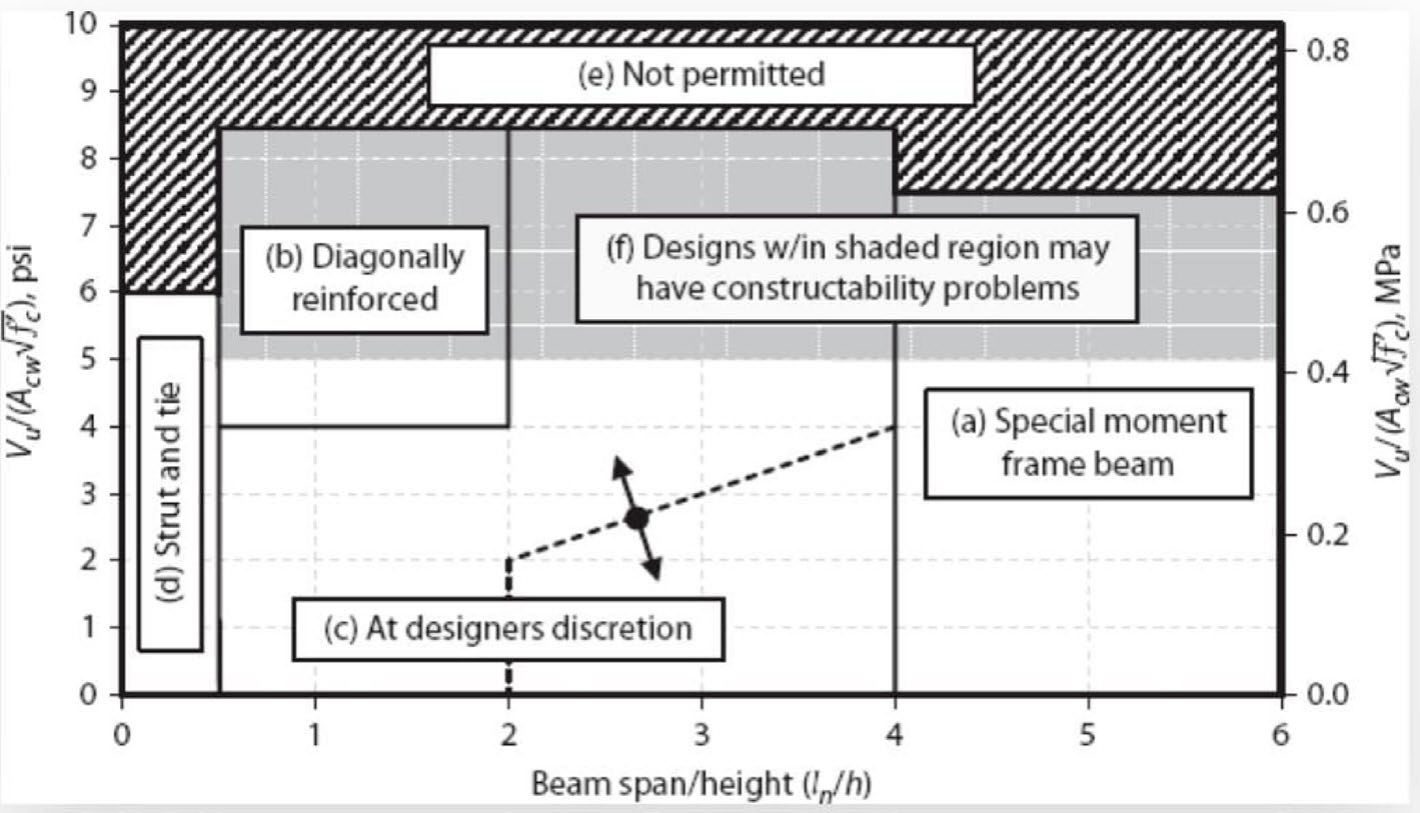
Coupling beam design guidelines as per ACI Code.

ACI-318^2^ specifies the use of conventional reinforcement (top and bottom bars with confinement stirrups) for CBs with a span-to-depth ratio greater than 4. For short coupling beams (ln/h < 2), the use of diagonally reinforced concrete—consisting of two intersecting cages of symmetrical diagonal reinforcement—is recommended. For intermediate beams (2 < ln/h < 4), the appropriate reinforcement detail is determined by the shear stress level, as illustrated in Fig. 1.

For seismic performance, diagonally reinforced beams consistently outperform conventional ones. Studies^3–12^ show they provide stable hysteretic behavior, higher ductility, and superior energy dissipation.

Research on reinforced concrete coupling beams focuses on two main goals: improving the seismic performance of conventional beams and simplifying the complex construction of diagonal reinforcement. Enhancements like U-type reinforcement at the beam edge with intermediate bars anchored into the walls^13^ effectively prevent brittle shear failure in conventional beams and was recommended as a viable construction method for achieving design strength. To reduce congestion in diagonal reinforcement, researchers have successfully tested alternatives like using diagonal steel plates^3^, high-strength materials^14^, partial substitution using a single diagonal bar and headed bars^15^, bundled bars with steel fibers^16^, and rhombic detail^12,17,18^. Innovative designs like the "Double-Beam" detail^19^ also show promise in achieving equal or better performance with simpler construction.

**Fig. 2:**
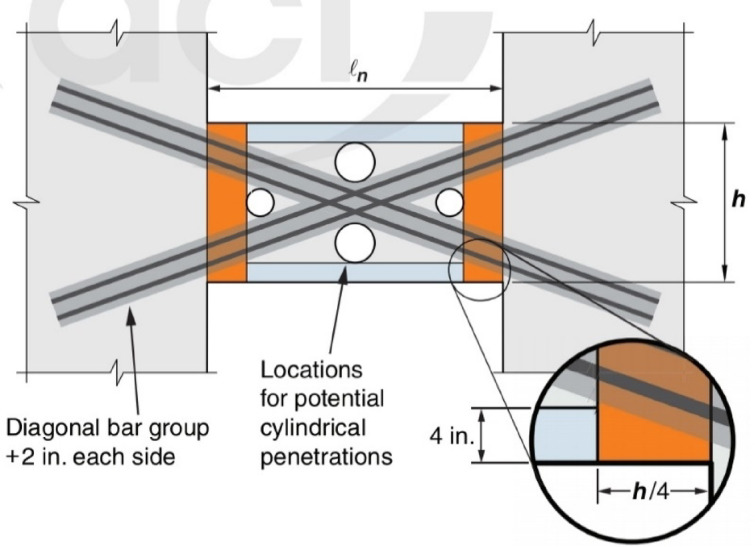
Coupling beam elevation showing prohibited regions for penetrations (shaded). *Note*: The figure illustrates four potential penetration locations, but only two are permitted in a single beam (Adapted from ACI 318–25^20^).

To address the impact of openings, ACI 318–25^20^ incorporates recommendations from Abdullah et al.^21^ for circular penetrations in diagonally reinforced CBs. These stipulate a maximum of two penetrations, with a diameter not exceeding h/6 or 150 mm. Penetrations must have specified clearances from diagonal bars, beam ends, top/bottom surfaces, and adjacent penetrations. Figure 2 illustrates the zones where penetration is prohibited as recommended in^20^. Research into the detrimental effects of service rectangular opening/s on performance of RCCBs has consistently shown that the location of the openings is a significant factor, concluding that openings positioned at the beam ends are substantially more detrimental to structural performance—causing notable reductions in stiffness and capacity- than those placed near the center of the span^22^. This has been further enhanced by another investigation^23^, which confirmed that a rectangular opening near the beam end leads to notable losses in strength and ductility while altering the failure mode. Importantly, the same study demonstrated that these negative effects can be successfully mitigated by reinforcing the opening with diagonal bars and using short stirrups in the beam sections above and below the opening. The additional reinforcement restored the structural behavior effectively, and brought the beam behavior closer to that of a solid one (without openings).

## Research significance

Coupling beams act as structural fuses within a coupled shear wall system. If these beams are overly rigid, they will undergo only limited rotations and dissipate little energy before failure. In such a case, the beams would fail before the wall piers, thus defeating their primary purpose. Therefore, appropriate reinforcement is essential to ensure a ductile behavioral response, allowing the beams to absorb significant energy before failure.

The routing of service ducts often requires penetrations through the web of coupling beams. These openings can weaken the beams and alter their structural behavior, leading to performance that differs from that of solid beams. However, current research on the structural performance of coupling beams with openings remains limited. In addition, existing studies have predominantly focused on diagonally reinforced beams, leaving a significant gap in understanding the interaction between web openings and alternative reinforcement detailing, such as conventional or rhombic layouts. Furthermore, prior experimental investigations have often been constrained by scope—either examining a limited number of beam sizes^22^ or a restricted range of opening locations^23^ —while existing guidance in^20^ is limited to openings of circular geometry. To address this research gap effectively, a broad parametric investigation is essential. However, conducting a comprehensive experimental program of such scope is prohibitively expensive, time-consuming, and often limited by the complexity of test setups and equipment constraints. There, this study verifies then employs a numerical approach using the finite element software ABAQUS. This methodology facilitates an efficient and detailed parametric investigation into the cyclic performance of short coupling beams with various reinforcement layouts (conventional, diagonal, and rhombic) and web openings. The primary objectives are to compare the seismic behavior of these systems and to evaluate the influence of opening size and location. The results are intended to provide insights to guide design engineers in making informed decisions regarding the integration of service openings in coupling beams with diverse reinforcement schemes when such penetrations are necessary.

## Verification models

A model verification study was conducted to validate the chosen modeling techniques, including element types, material constitutive models, boundary conditions, and convergence criteria. To this end, four experimentally tested specimens were simulated to evaluate the capability of the non-linear FE program (ABAQUS) in accurately capturing the behavior of reinforced concrete coupling beams (RCCBs).

The following sections describe the selected specimens and their experimental setups, detail the modeling methodology, and present a comparison between the analytical and experimental results.

### Description of the proposed verification specimens

Four specimens (P01, P02, P05, and P07) from Galano and Vignoli^18^ were selected for model verification. All specimens had a width of 150 mm, with a depth of 400 mm and a length of 600 mm. The reinforcing steel had an average yield strength (fy) of 567 MPa, with individual values ranging from 528 to 611 MPa. The average concrete compressive strengths were 48.9 MPa, 44.5 MPa, 39.9 MPa, and 54.0 MPa for specimens P01, P02, P05, and P07, respectively.

Specimens P01 and P02 featured conventional reinforcement detailing, while P05 and P07 were diagonally reinforced without confinement around the diagonal bars. The loading protocols differed: P01 and P05 were tested under monotonic loading, whereas P02 and P07 were subjected to cyclic loading.

**Fig. 3:**
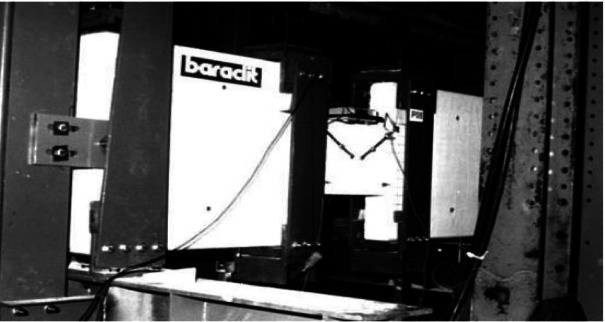
Experimental test setup (after Galano and Vignoli^18^).

**Fig. 4:**
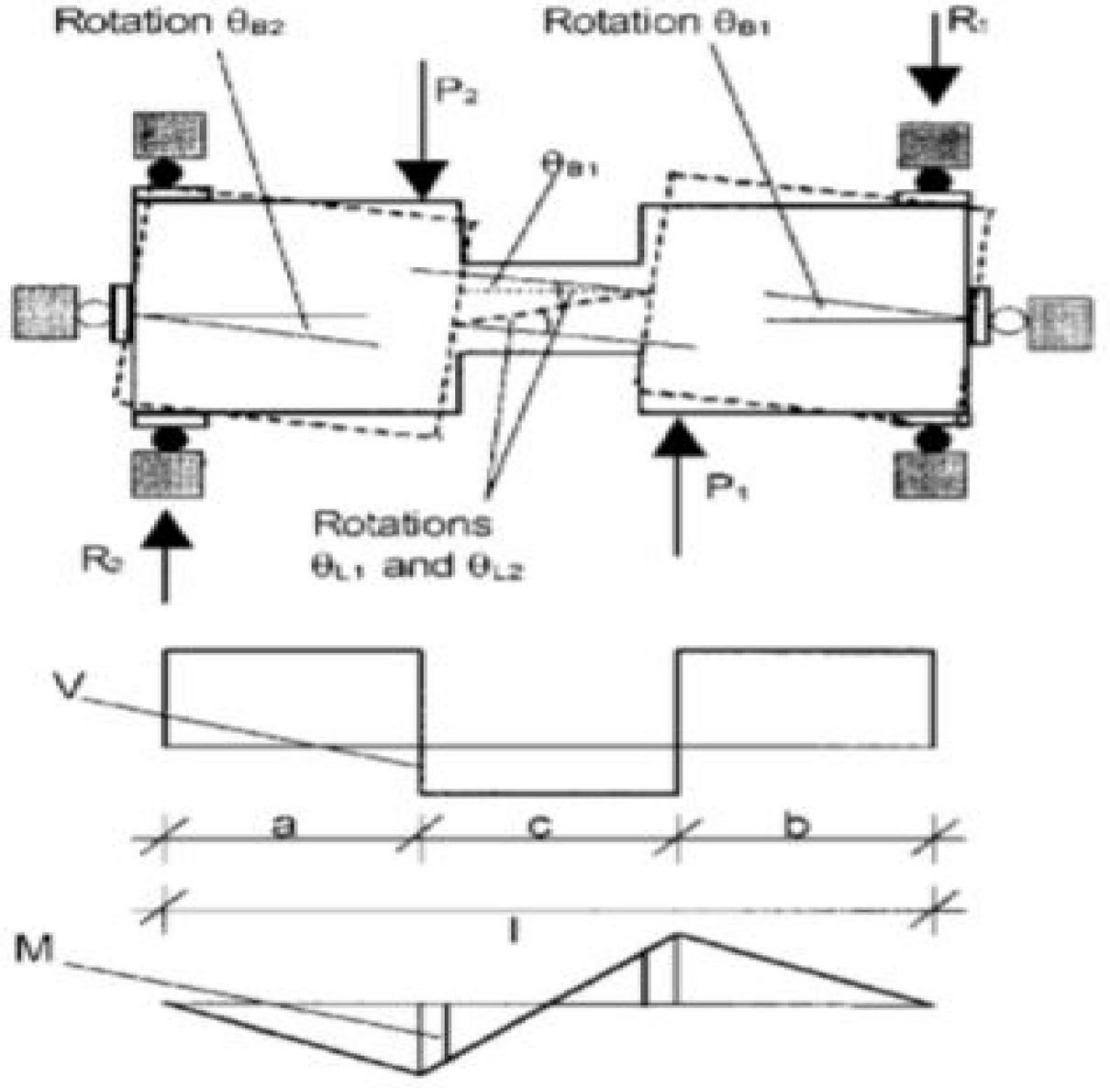
Resulting internal forces: shear and bending moment diagram (after Galano and Vignoli^18^).

**Fig. 5:**
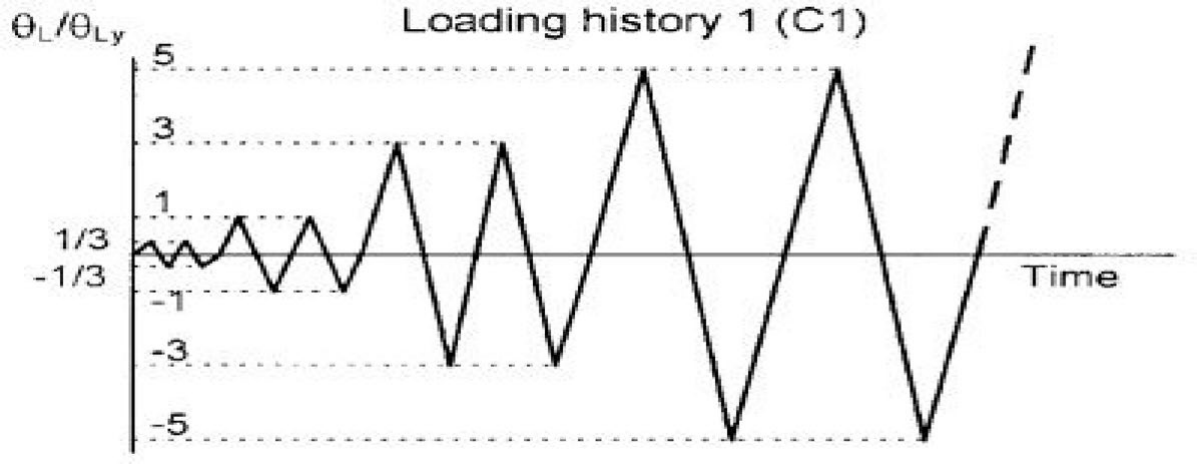
Loading history for specimens P02 and P07.

The experimental setup, illustrated in Figs. 3, 4, and 5, utilized two lateral stiff blocks at the ends of the coupling beams to simulate the boundary conditions provided by the adjacent walls. Each block was restrained by three rollers to prevent horizontal movement and vertical movement at the supports. Loading was applied by two actuators, one on each block, moving in opposite directions to induce anti-symmetric bending and maintain a constant shear force along the beam’s span. Figure 3 shows the test setup, Fig. 4 diagrams the resulting internal forces, and Fig. 5 presents the applied load histories.

## Finite element modelling

Based on the central objectives of this research, three-dimensional finite element models of the reinforced concrete coupling beams (RCCBs) were developed using the ABAQUS software.

### Element types

The concrete beams and boundary blocks were modelled using 8-noded, 3D solid elements with reduced integration (C3D8R). Both the longitudinal reinforcement and stirrups were modelled using 2-noded, 3D truss elements (T3D2). The reinforcement was fully embedded into the concrete mesh, assuming a perfect bond between the two materials.

### Material properties

Using the appropriate material models plays an important role in accurately evaluating the seismic performance of reinforced concrete (RC) members^24^. Due to the limitations of a smeared concrete model under cyclic loading, the Concrete Damaged Plasticity (CDP) model was employed. This model, originally developed by Lubliner et al. (1989) and later enhanced by Lee and Fenves (1998), defines the inelastic behavior of concrete. The elastic phase is characterized by the modulus of elasticity and Poisson’s ratio. The plastic response is governed by five key parameters: the dilation angle (Ѱ), flow potential eccentricity (ε), the ratio of initial biaxial to uniaxial compressive yield stress (${\sigma }_{b0}$ / ${\sigma }_{c0}$), the ratio of the second stress invariant on the tensile meridian to that on the compressive meridian (${K}_{c}$), and Viscosity parameter (µ).

While the dilation angle (Ψ) is known to significantly influence simulation outcomes^25–28^, there is no consensus on its specific value. The analysis in this study found that a dilation angle within the range of 30° to 40° produced the most acceptable agreement with the experimental data. Other parameters are set based on common practices: eccentricity (ε) is calculated from the tensile/compressive strength ratio, but is typically assumed as 0.1; the strength ratio (σb0/σc0) uses the ABAQUS default of 1.16; and the Kc ratio is set to the recommended 2/3. Finally, to ensure convergence without compromising accuracy, a small viscosity parameter of 1 × 10⁻⁶ is adopted based on^27^. All final parameter values are listed in Table 1.

**Table 1:** Parameters for the concrete damaged plasticity (CDP) model.

Parameter	Value
Poisson’s ratio (υ)	0.2
dilation angle (Ѱ)	${35}^{\mathrm{o}}$
Eccentricity (ε)	0.1
(${\sigma }_{b0}$ / ${\sigma }_{c0}$)	1.16
${K}_{c}$	0.667
Viscosity parameter (µ)	1e-6

The evolution of the yield (or failure) surface is governed by two hardening variables: the tensile equivalent plastic strain ${\varepsilon }_{t}^{\sim pl}$ and the compressive equivalent plastic strain and ${\varepsilon }_{c}^{\sim pl}$. These variables are linked to the failure mechanisms under tensile and compressive loading, respectively.

The total strain tensor ε is decomposed into an elastic component ${\varepsilon }^{el}$ and a plastic component ${\varepsilon }^{pl}$.

These plastic strain components are related to the degradation of the material’s elastic stiffness, which is represented by the damage parameters dt​ and dc​.

1 $$\varepsilon = { }\varepsilon^{el} + { }\varepsilon^{pl}$$

2 $$\sigma = D^{el} :\left( {\varepsilon - \varepsilon^{pl} } \right)$$

3 $$\overline{\sigma } = D_{o}^{el} :\left( {\varepsilon - \varepsilon^{pl} } \right)$$

4 $$D^{el} = \left( {1 - d} \right)D_{o}^{el}$$

Therefore, the stress–strain relationship is governed by the scalar damage elasticity equation:

5 $$\sigma = \left( {1 - d} \right)D_{o}^{el} :\left( {\varepsilon - \varepsilon^{pl} } \right)$$

Where:

- σ = stress in concrete (MPa).
- ${\mathrm{D}}_{\mathrm{o}}^{\mathrm{e}\mathrm{l}}$ = initial (undamaged) elastic modulus of the material (MPa).
- d = scalar stiffness degradation variable.
- ε = total strain.
- $${\epsilon}^{pl}$$ = plastic strain.

#### Boundary conditions, loading, and meshing

Modeling all components of the experimental setup—such as steel frames, plates, and bolts—was found to be computationally prohibitive, requiring excessive time for both model preparation and solution. Therefore, a simplified model was adopted to reduce preprocessing effort and solver time.

**Fig. 6:**
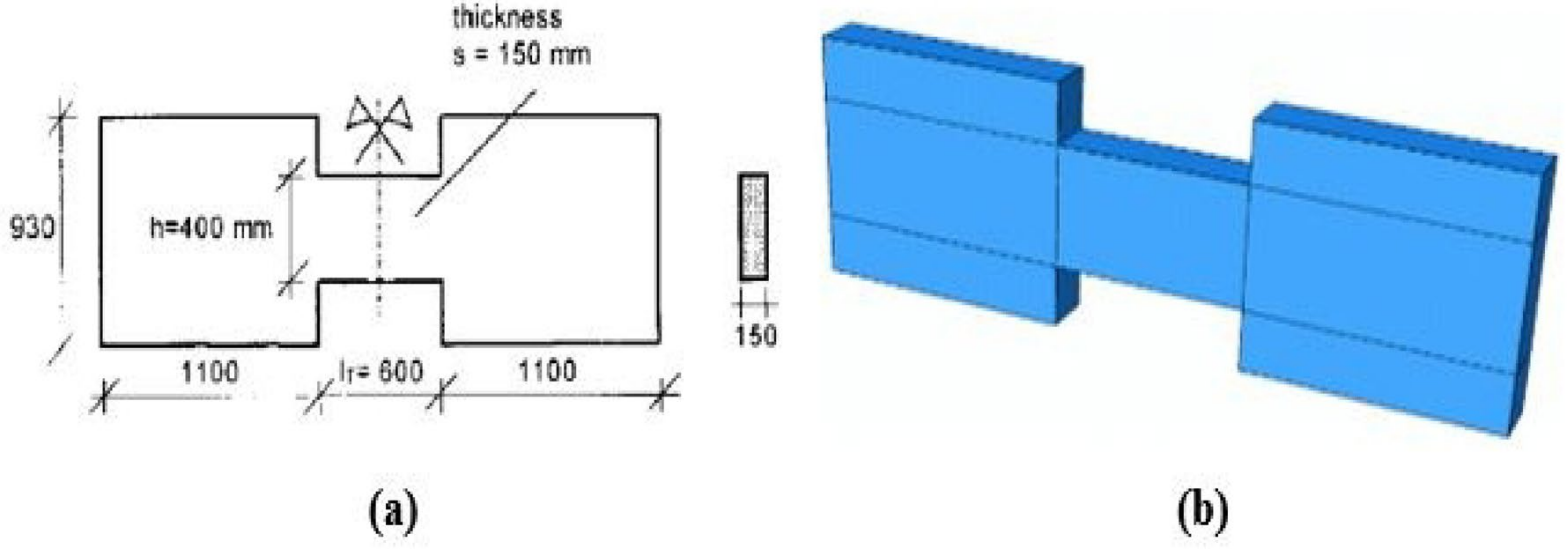
Geometry and dimensions of the four verification specimens: (**a**) dimensions (units in mm) and (**b**) the corresponding finite element model.

**Fig. 7:**
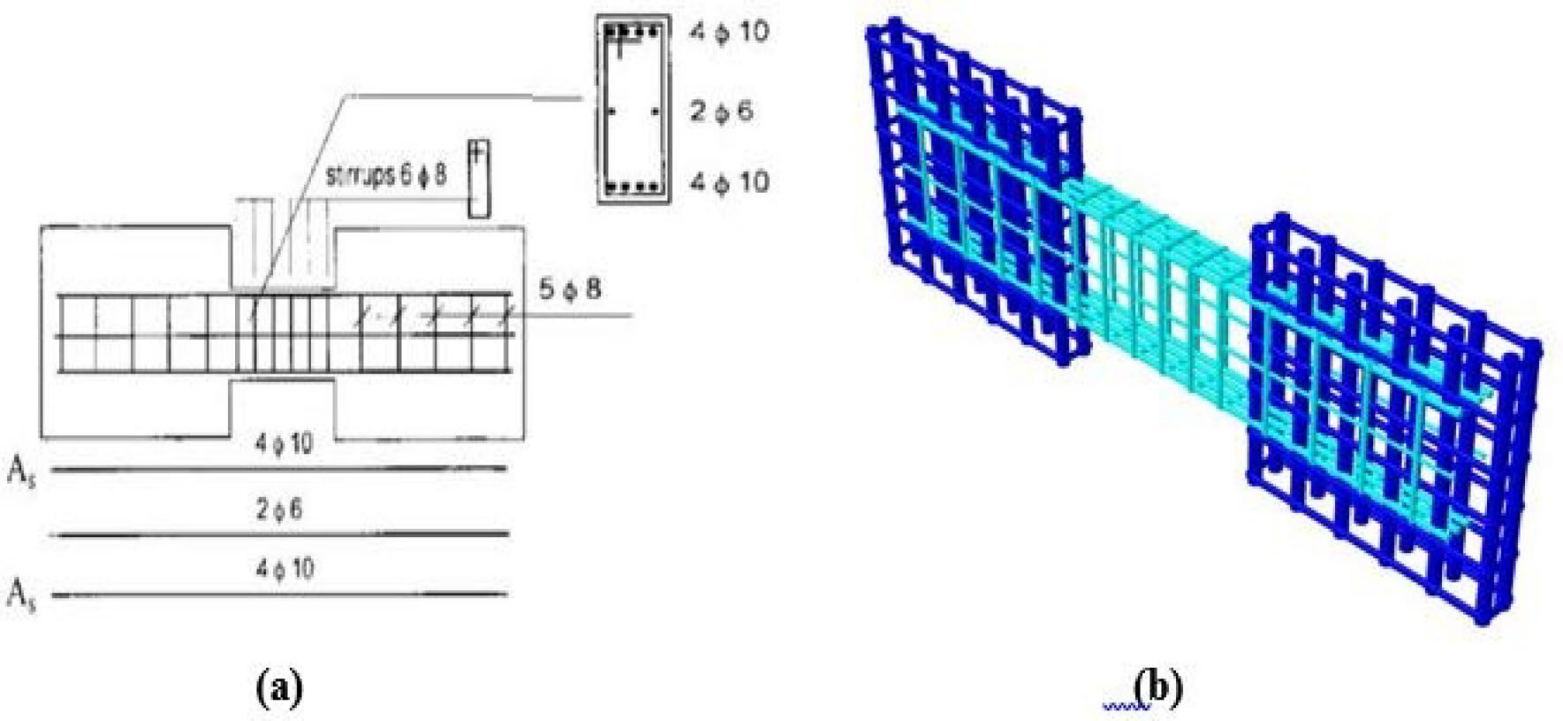
Reinforcement detailing for conventional specimens (P01 and P02): (**a**) as-built experimental layout and (**b**) as-modeled finite element representation.

**Fig. 8:**
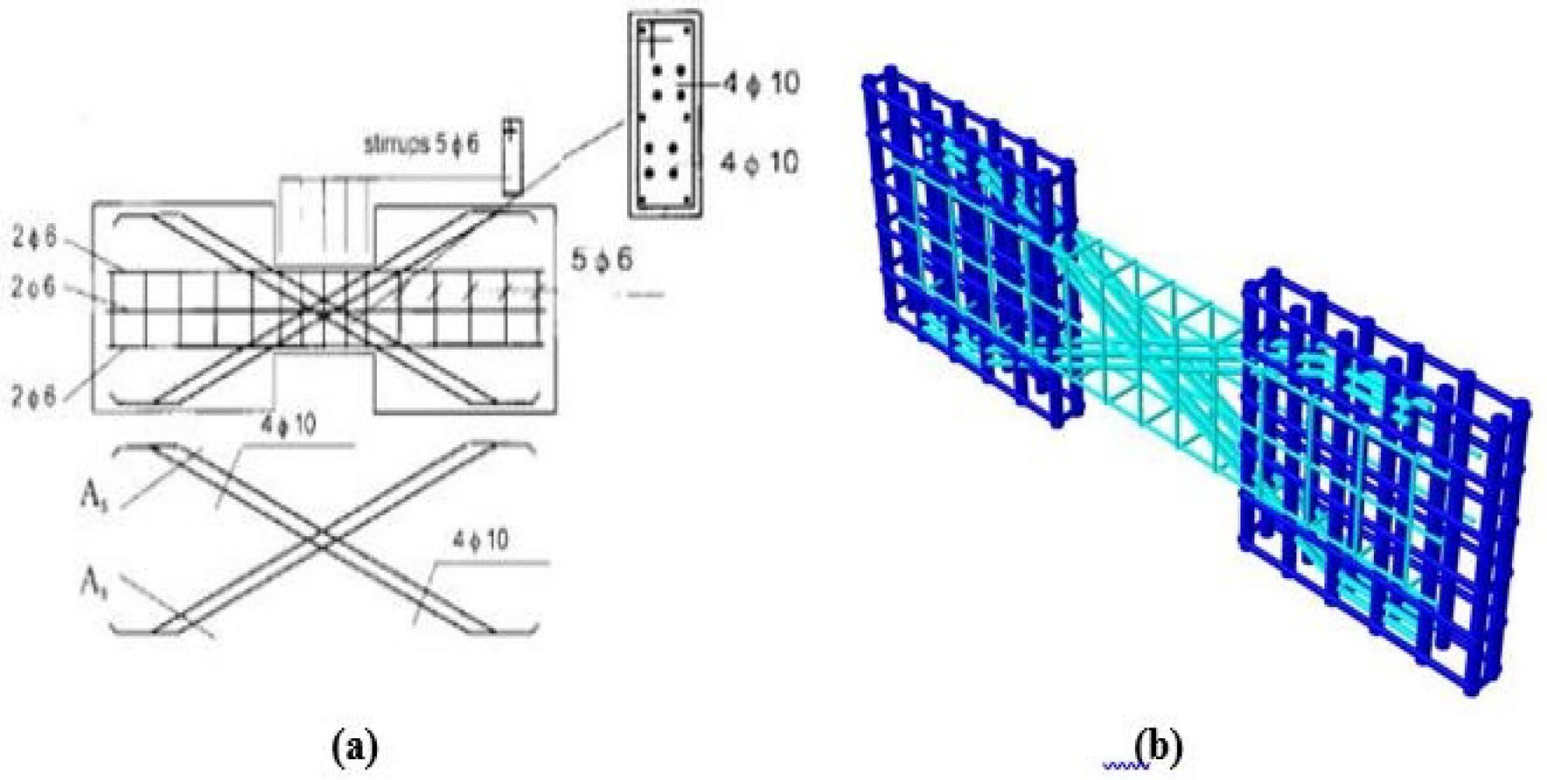
Reinforcement detailing for specimens with diagonal bars (P05, P07): (**a**) as-built experimental layout and (**b**) as-modeled finite element representation.

**Fig. 9:**
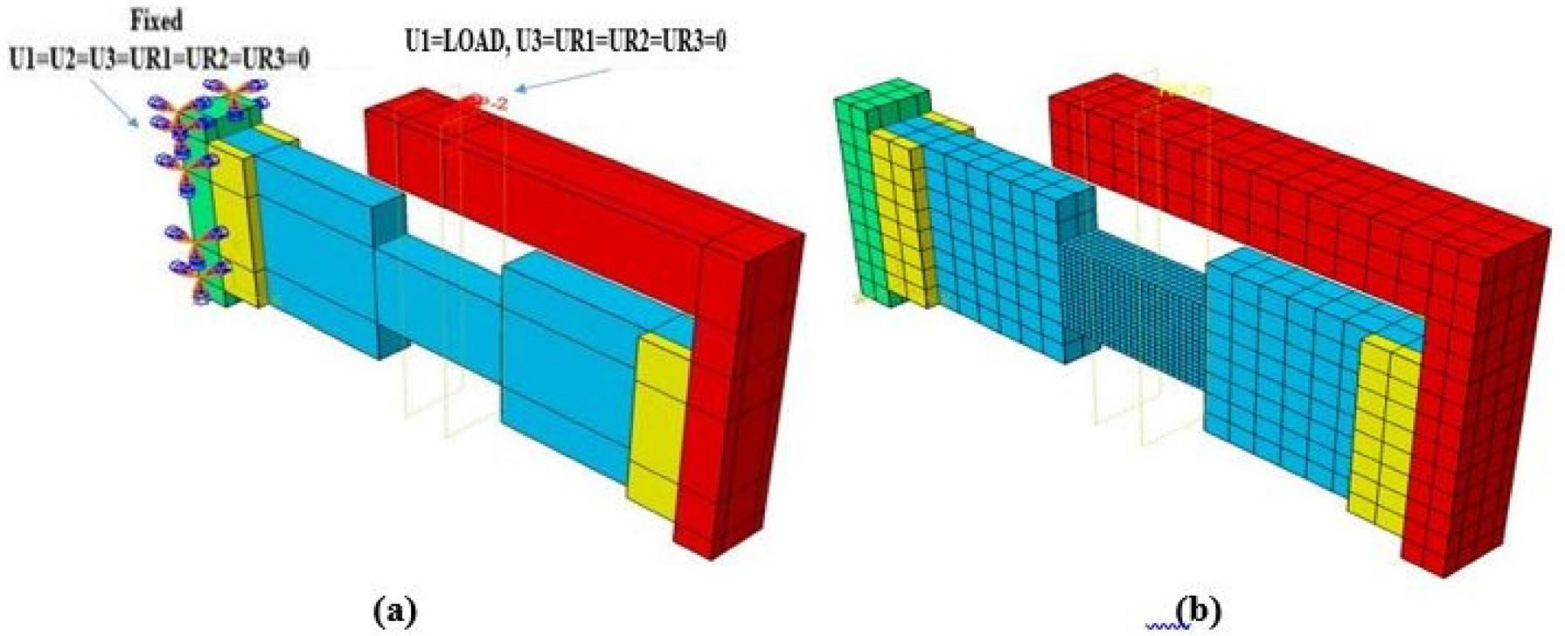
Finite element modeling setup: (**a**) applied loads and boundary conditions and (**b**) configuration of the computational mesh.

In this simplified approach, the complex setup was replaced with a very stiff loading plate, tied to the surface of the loading block. The elastic modulus and dimensions of this plate were selected to ensure load was transferred to the coupling beam without inducing local rotation. Similarly, a rectangular steel plate was tied to the support block to represent fixed constraints. Additional plates were added at the ends of both blocks to prevent stress concentrations near the loading and support interfaces. A mesh sensitivity analysis was conducted to balance accuracy and computational cost. As is typical in FE analysis, finer meshes improve results but increase solving time. After several trials, a mesh size of 25 × 25 mm for the coupling beam and 100 × 100 mm for the boundary blocks and steel plates was selected, providing acceptable accuracy within a reasonable solution time. The specimen geometry, reinforcement details, the proposed FE model setup, boundary conditions, and mesh are illustrated in Figs. 6, 7, 8 and 9.

#### Validation of the structural model

The finite element model (FEM) was validated through a comprehensive comparison with experimental data. Key structural characteristics- including damage and crack pattern, failure mode, and the overall shear versus chord rotation hysteretic response- were analyzed.

### Damage and crack pattern comparison

**Fig. 10:**
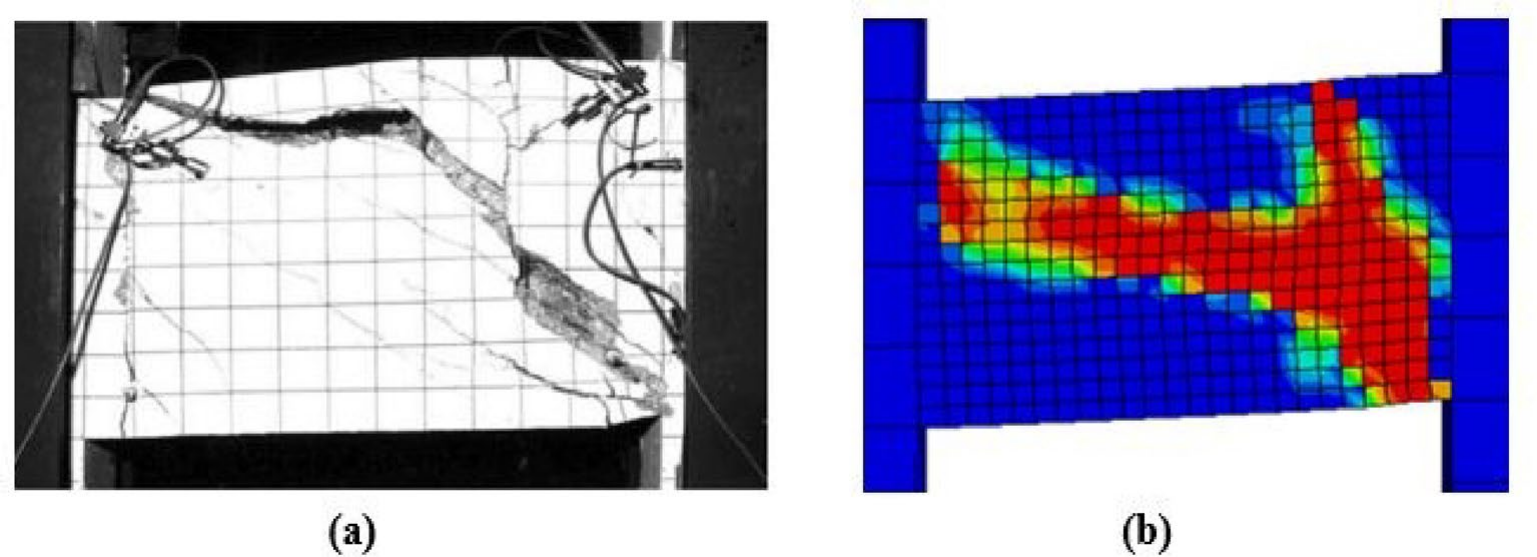
Damage in Specimen P01: (**a**) experimental and (**b**) FEM.

**Fig. 11:**
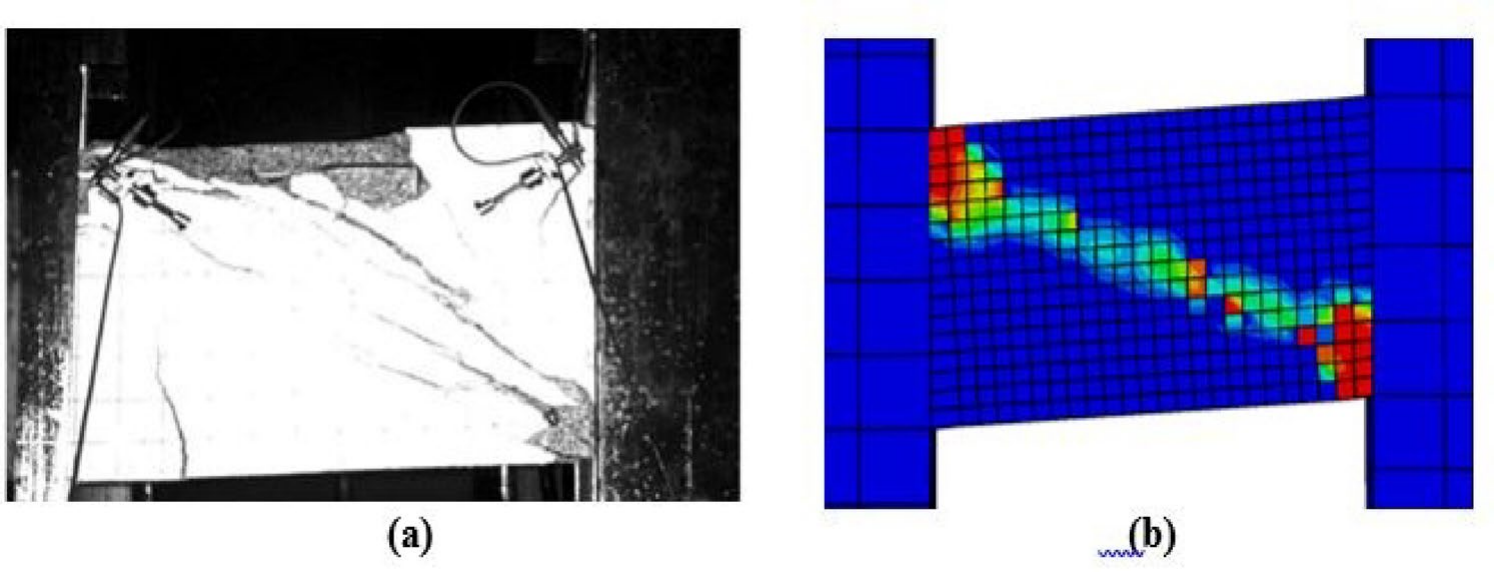
Damage in Specimen P05: (**a**) experimental and (**b**) FEM.

**Fig. 12:**
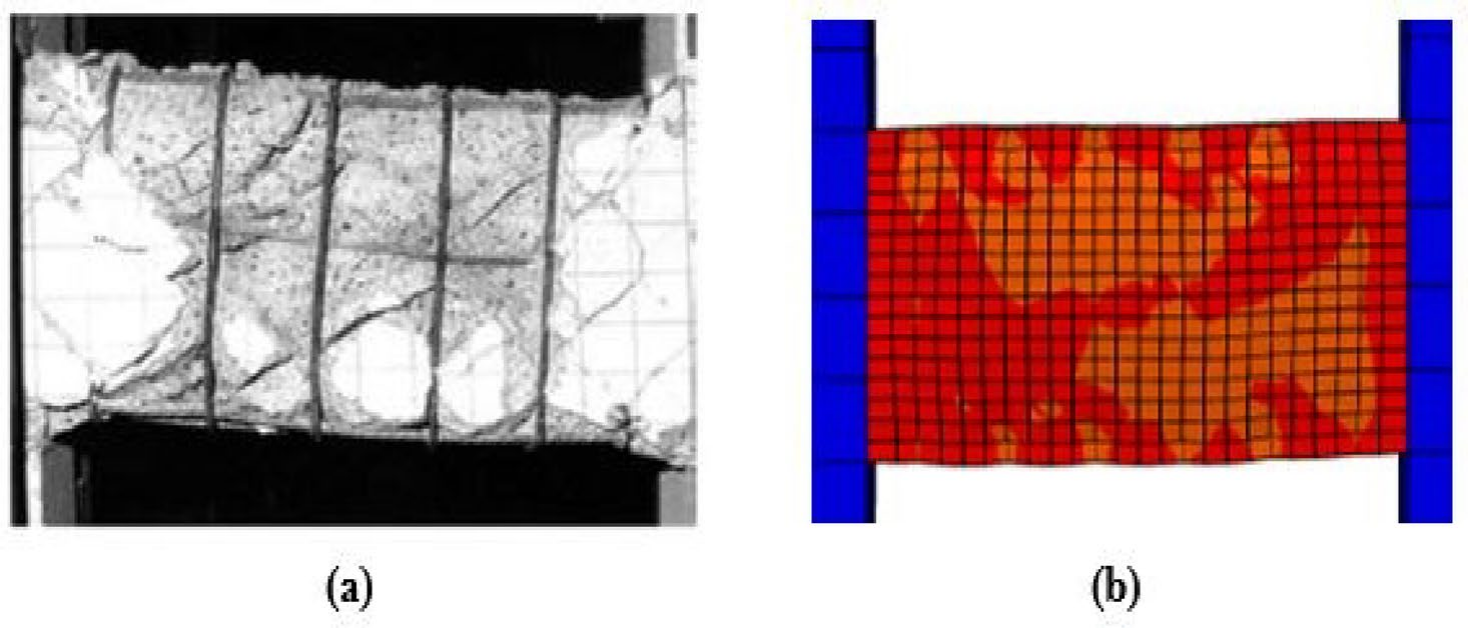
Damage in Specimen P02: (**a**) experimental and (**b**) FEM.

**Fig. 13:**
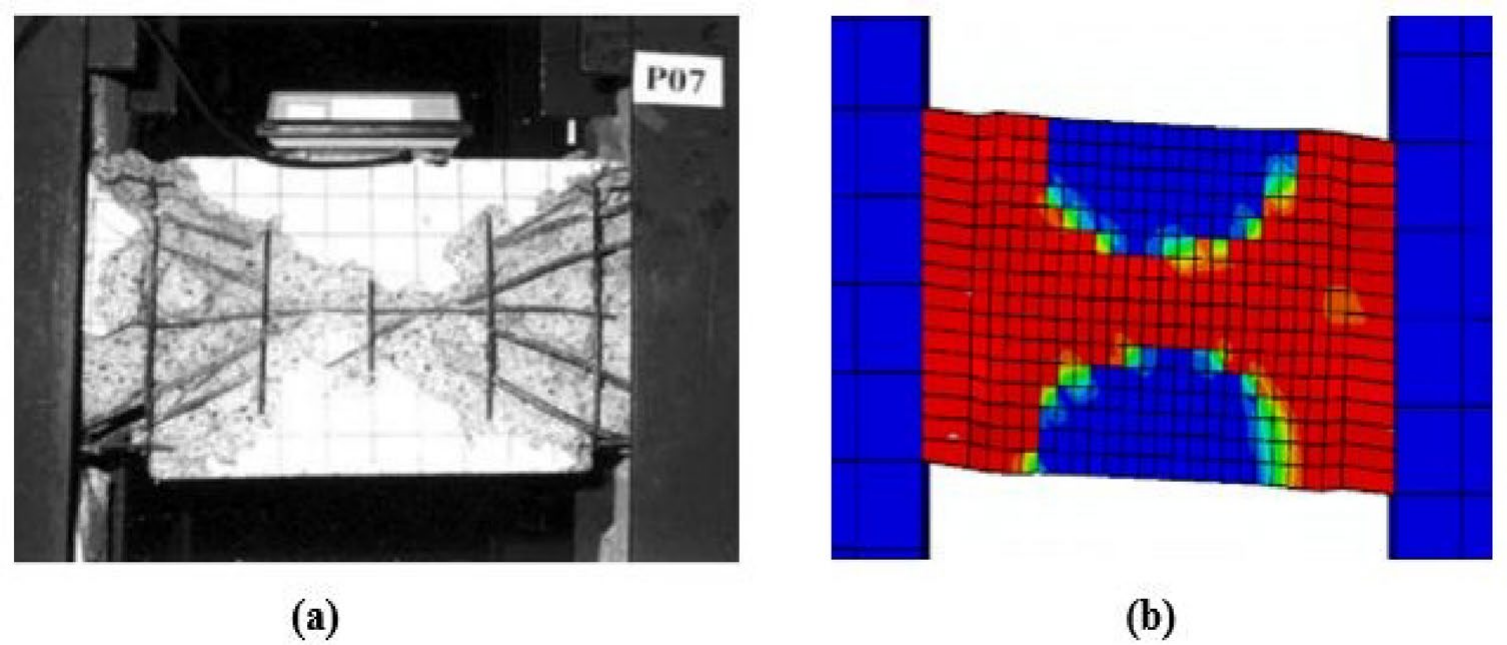
Damage in Specimen P07: (**a**) experimental and (**b**) FEM.

Figures 10, 11, 12 and 13 present a visual comparison of the final damage states for Specimens P01, P05, P02, and P07, respectively. In each figure, part (a) shows the damage from laboratory testing, while part (b) shows the simulated damage from the numerical analysis.

- For the specimens subjected to monotonic loading (P01, P05), the finite element model demonstrates strong agreement with the experimental damage patterns. As illustrated in Figs. 10 and 11, both the experimental and numerical results exhibit primary diagonal shear cracking. The FEM accurately captures the crack inclination, propagation path, and the resulting shear-dominated failure mode.
- Under cyclic loading, damage progression varied significantly with reinforcement detailing. As shown in Fig. 12, the conventionally reinforced specimen exhibited distributed surface cracks, which the finite element model accurately reproduced. In contrast in Fig. 13, the diagonally reinforced specimen (P07) showed damage concentrated along the primary diagonal. The numerical simulation successfully captured this distinct pattern, validating the model’s ability to simulate the improved cyclic performance of this detailing.
- In all cases, the FEM demonstrates a strong practical agreement with the experimental crack patterns and failure mode confirming that the model’s constitutive laws and element formulations are appropriate for simulating different failure mechanisms of coupling beams.

### Shear-rotation hysteretic response

**Fig. 14:**
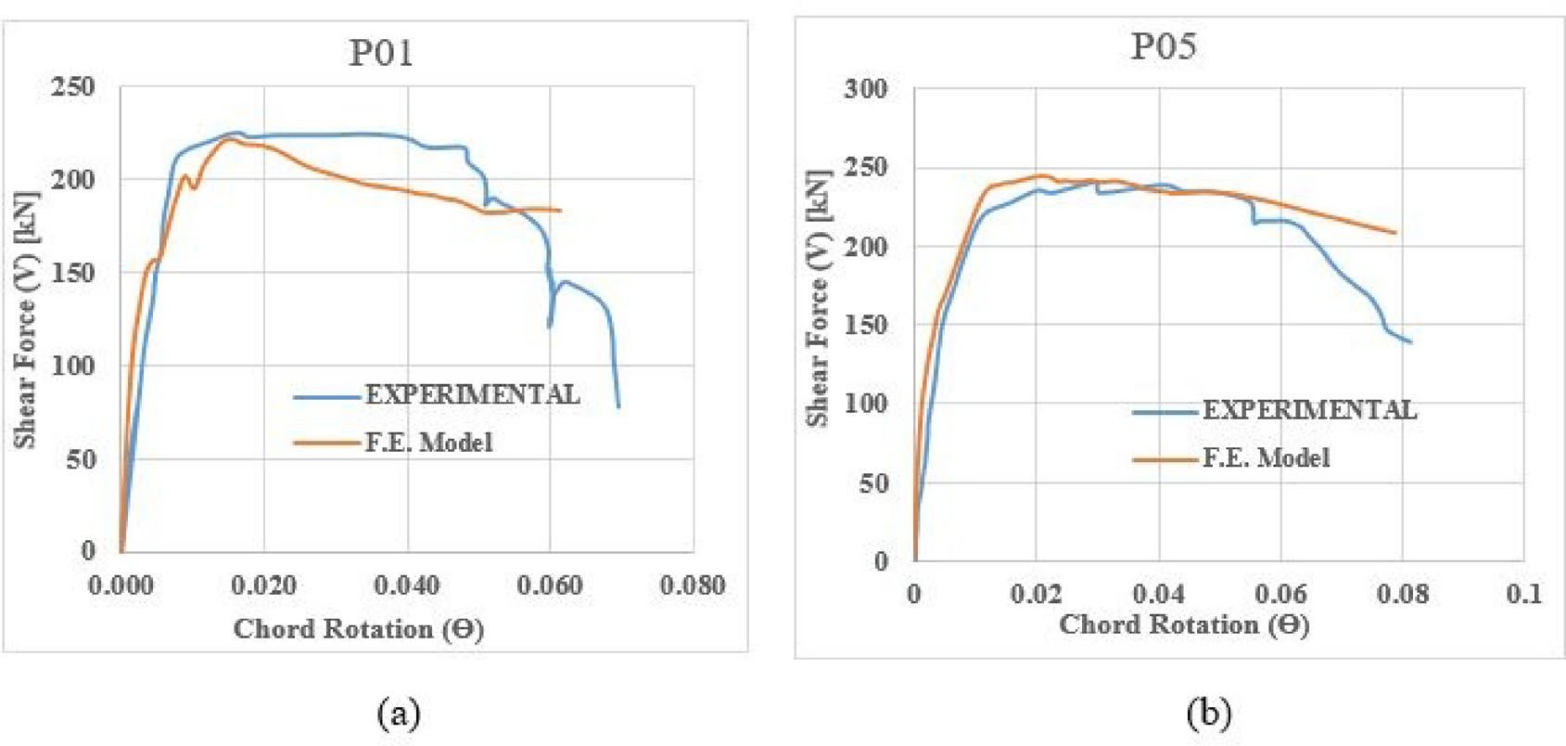
Shear-chord rotation response under monotonic loading: (**a**) Specimen P01 and (**b**) Specimen P05.

**Fig. 15:**
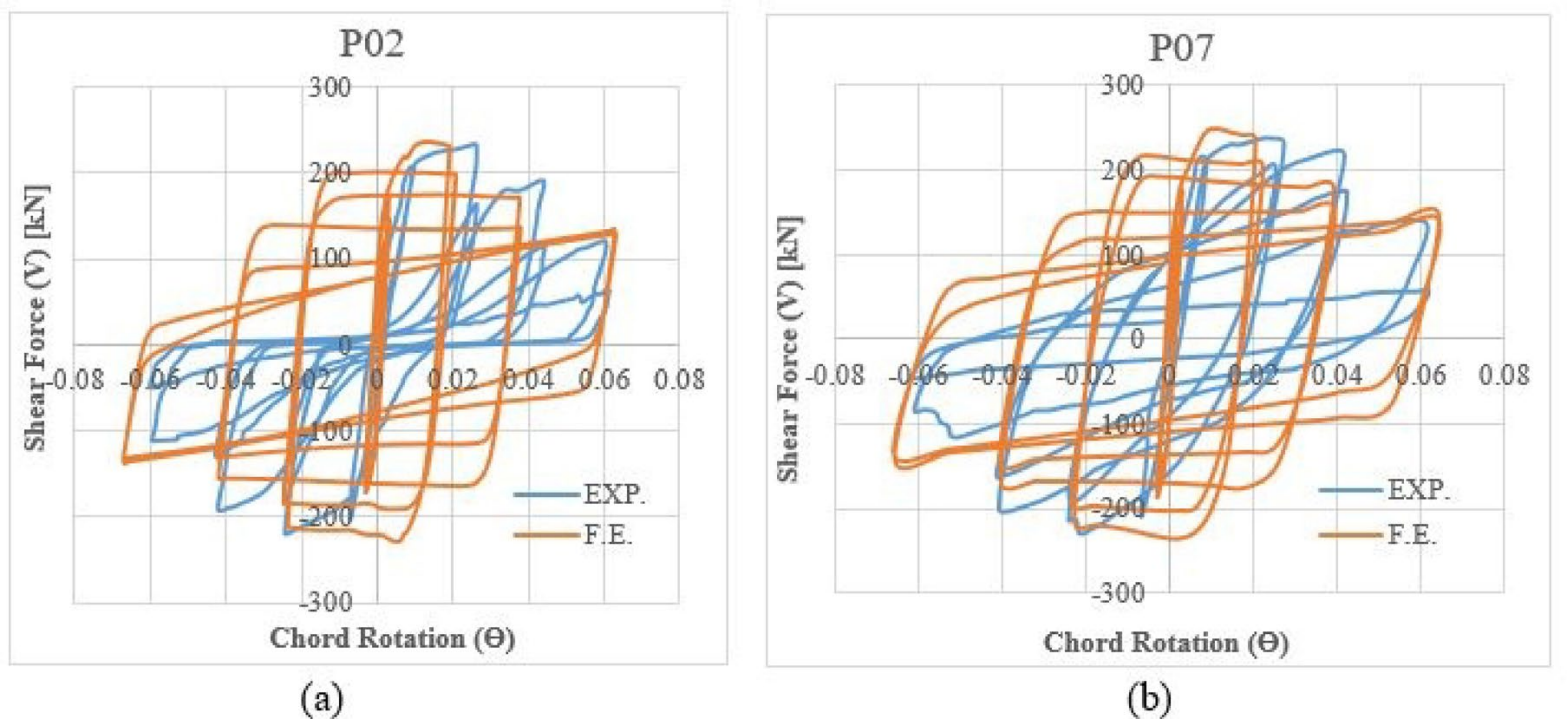
Shear-chord rotation response under cyclic loading: (**a**) Specimen P02 and (**b**) Specimen P07.

Figures 14 and 15 compare the experimental and analytical shear-chord rotation responses under monotonic and cyclic loading, respectively.

- For the conventionally reinforced specimen P01 (Fig. 14a), both the experimental and analytical curves exhibit a sharp, well-defined peak. The model accurately predicts this maximum strength, after which a rapid, brittle decline in load-carrying capacity is observed. This post-peak response is characteristic of a shear-critical failure. In contrast, the peak strength for specimen P05 (Fig. 14b), which features enhanced reinforcement detailing, is captured with equal accuracy by the model. The notable difference, however, lies in the post-peak behavior. While a strength decay is present, the FEM curve suggests a more gradual degradation compared to P01.
- For the cyclically loaded specimens, the FEM hysteresis loops closely match the experimental data in both envelope and path, as shown in Fig. 15. The FEM hysteresis loops demonstrate close agreement with their experimental counterparts, accurately reproducing both the overall envelope and the detailed loading/unloading paths. This validation confirms the model’s capability to capture essential cyclic characteristics- including pinching behavior, progressive strength degradation, and energy dissipation capacity- that are fundamental for evaluating seismic performance.

### Conclusion of validation

The consistent correlation between the numerical and experimental results—from visual damage patterns to quantitative load-deformation responses—confirms the validity of the developed finite element model. The model effectively predicts the different failure mechanisms of conventional and diagonal detailing. Furthermore, it can not only replicate the final damage state but also accurately capture essential cyclic characteristics- including pinching behavior, progressive strength degradation, and energy dissipation capacity- that are fundamental for evaluating seismic performance. Therefore, this validated FEM can be used with confidence for subsequent parametric studies or to analyze structural configurations not explicitly tested in the laboratory program.

## Research plan

A parametric study was conducted using a set of twelve finite element models of reinforced concrete coupling beams (RCCBs). The models were organized into four groups based on their reinforcement scheme:

- Group C: Conventional reinforcement.
- Group DC: Diagonally confined reinforcement.
- Group R1: Rhombic configuration 1.
- Group R2: Rhombic configuration 2.

The specimen matrix was designed as follows:

- Groups DC and R1 each contained three specimens: one solid beam, one with an edge opening (EOp) located 37.5 mm from the support at the beam’s mid-height, and one with a central opening (COp) at the beam’s mid-span and bottom.
- Group C included an additional specimen with a different opening size at the mid-span.
- Group R2 contained two specimens: one solid and one with a mid-span opening.

All specimens had identical overall dimensions of 200 mm in width and 400 mm in both depth and length, resulting in a span-to-depth ratio (ln/h = 1). The opening size was selected based on the usually utilized sizes in practical designs to balance the beam strength against the need for service ducts. This practically feasible maximum size was kept constant across all reinforcement layouts for a controlled comparative study. Consequently, A single rectangular opening, measuring 50 mm in width and 100 mm in height, was incorporated into the beam models. This corresponds to an opening length-to-beam span ratio lo/l = 0.125 and an opening height-to-beam depth ratio ho/t = 0.25, representing a geometrically significant penetration. For one specimen, (C-COp2), the dimensions of opening were reversed from (lo/l = 0.125 and ho/t = 0.25) to (lo/l = 0.25 and ho/t = 0.125) to investigate the effect of opening orientation.

**Fig. 16:**
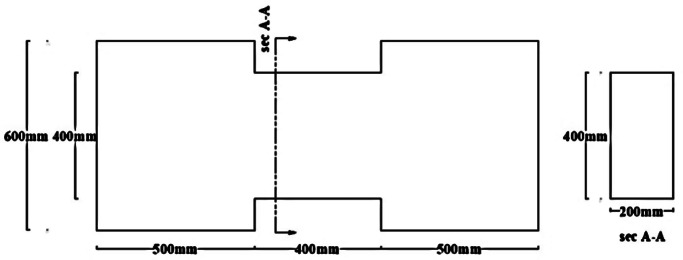
Geometry of the solid specimens: C-S, DC-S, R1-S, and R2-S.

**Fig. 17:**
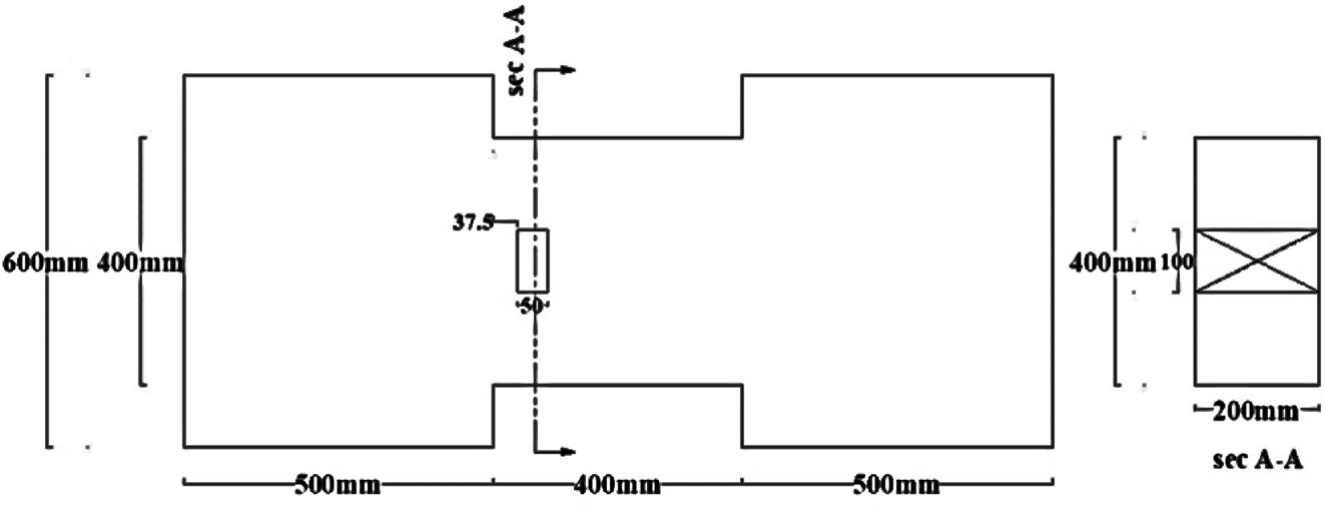
Geometry of specimens that have edge openings: C-EOp, DC-EOp, and R1-EOp.

**Fig. 18:**
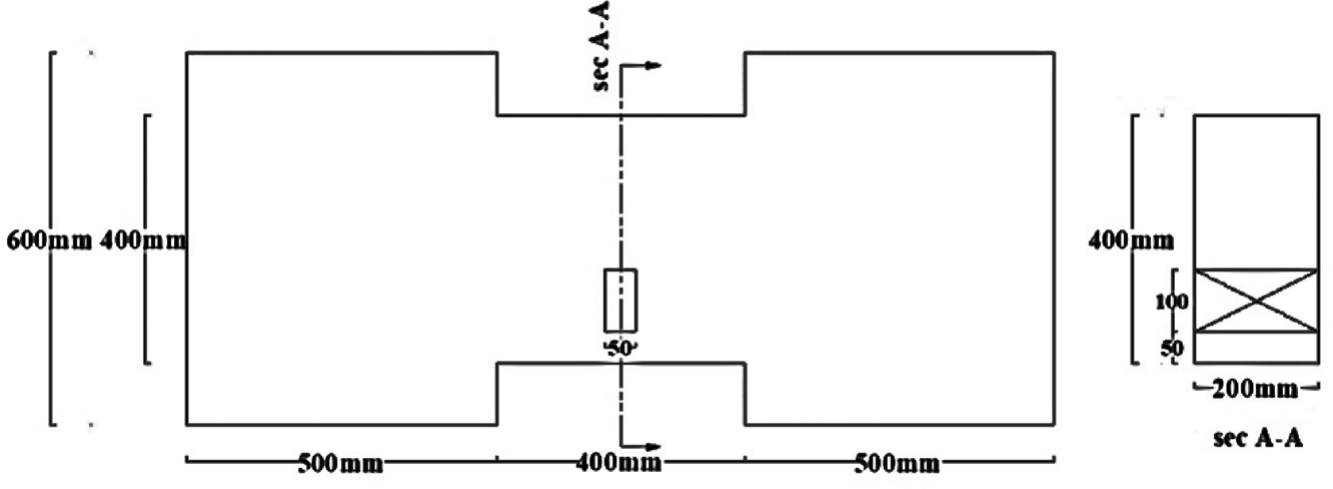
Geometry of specimens that have mid-span openings: C-COp1, DC-COp, R1-COp, and R2-COp.

**Fig. 19:**
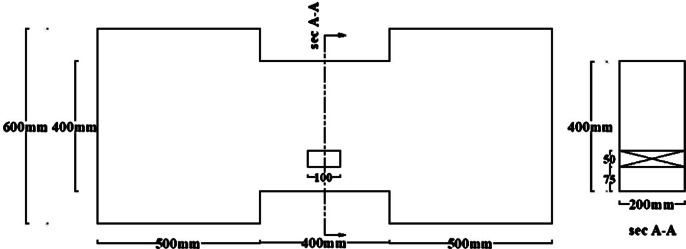
Geometry of specimen C-COp2.

**Fig. 20:**
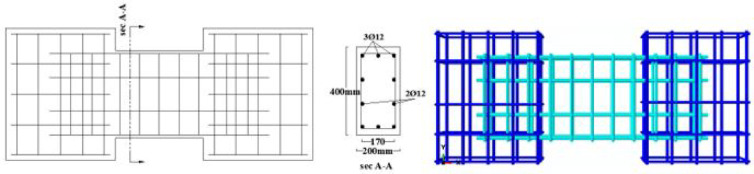
Reinforcement details of group (C): C-S, C-EOp, C-COp1, and C-COp2.

**Fig. 21:**
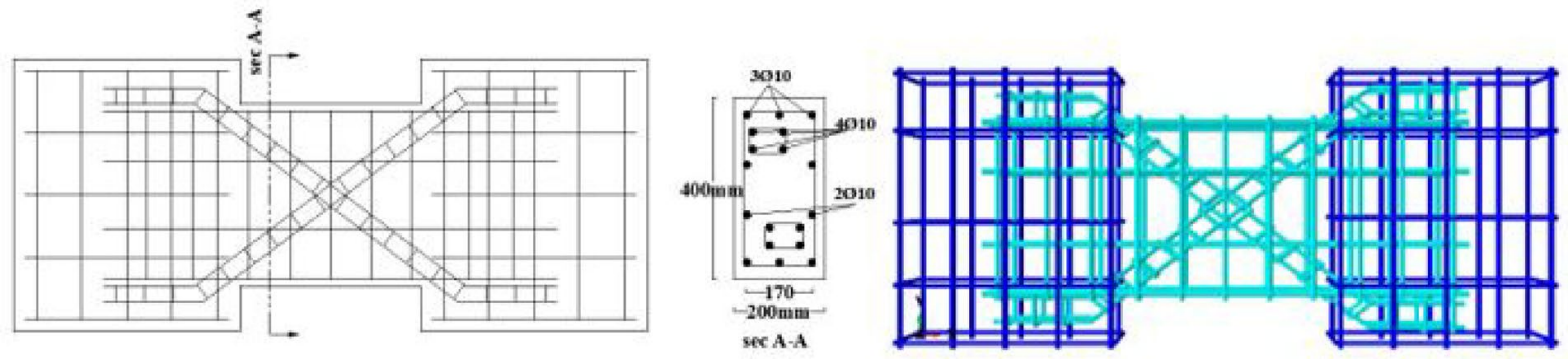
Reinforcement details of group (DC): DC-S, DC-EOp, and DC-COp.

**Fig. 22:**
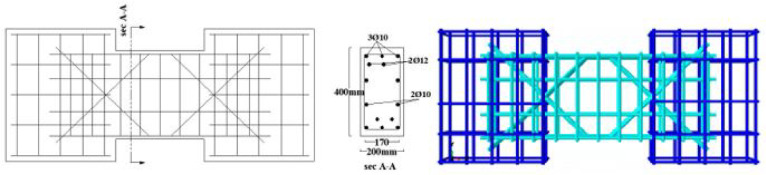
Reinforcement details of group (R1): R1-S, R1-EOp, and R1-COp.

**Fig. 23:**
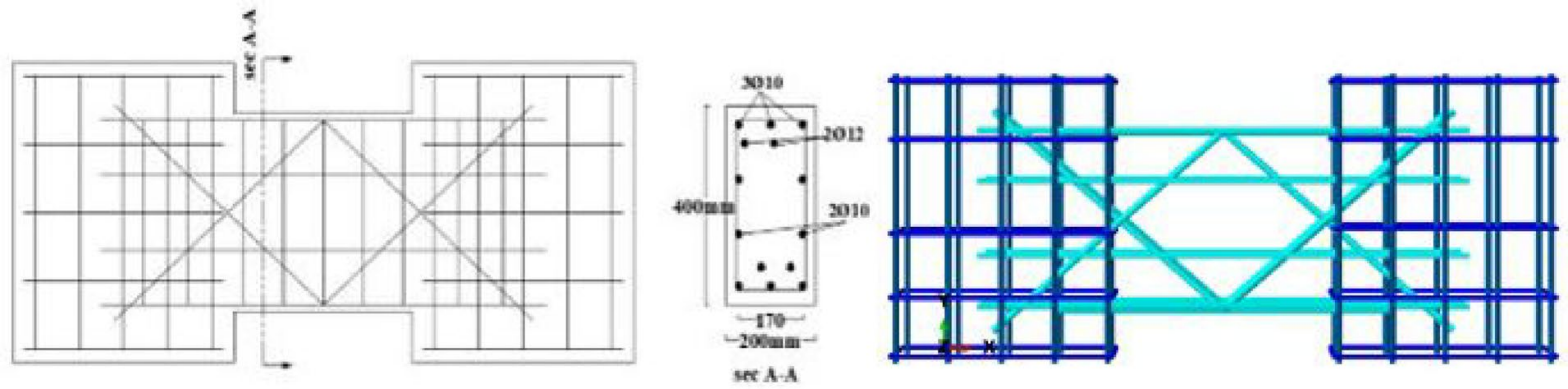
Reinforcement details of group (R2): R2-S and R2-COp.

The material properties were constant across all models, with a concrete compressive strength $f_{c}{\prime}$ of 35 MPa and a Poisson’s ratio of 0.20. The solid specimens were designed for a nominal shear stress (q) ranging from 0.30 to 0.38 $\sqrt {f_{c} } {^{\prime}}$ (MPa), calculated according to ACI 318–19^2^. The location of opening and reinforcement details for all specimens are summarized in Tables 2 and 3 and the geometry and reinforcement details are illustrated in Figs. 16, 17, 18, 19, 20, 21, 22 and 23. It is important to note that this study is limited to short, shear-critical RC coupling beams with span-to-depth ratios (l/h) of around 1.0, constructed using normal-strength concrete.

**Table 2:** Summary of analyzed specimens.

Group Name	Description	Beam No	Opening Location in longitudinal direction of the beam (S)	Opening Location in transverse direction of the beam (Z)
C	Conventional beam (with top and bottom reinforcement)	C-S	―	―
		C-EOp	Spacing of 37.5 mm from the fixed support	At the mid height
		C-COp1	At the mid span	Bottom
		C-COp2	At the mid span	Bottom
DC	Diagonally confinement cages	DC-S	―	―
		DC-EOp	Spacing of 37.5 mm from the fixed support	At the mid height
		DC-COp	At the mid span	Bottom
R1	Rhombic configuration1	R1-S	―	―
		R1-EOp	Spacing of 37.5 mm from the fixed support	At the mid height
		R1-COp	At the mid span	Bottom
R2	Rhombic configuration2	R2-S	―	―
		R2-COp	At the mid span	Bottom

**Table 3:** Reinforcement details for the analyzed specimens.

Group Name	Longitudi-nal Bars (Top and bottom)	Intermedi-ate Bars	Diag-onal Bars	α	Vertical stirrups	Diago-nal stirrups	${f}_{yl}$	${f}_{yd}$	${f}_{yst}$
C	3Ø12	2 Ø12 @110 mm	―	―	Ø10 @ 90 mm	―	400	―	360
DC	3Ø10	2 Ø10 @110 mm	4 Ø10	37°	Ø10 @ 90 mm	Ø10 @ 65 mm	360	360	240
R1	3Ø10	2 Ø10 @110 mm	2 Ø12	44°	Ø10 @ 90 mm	―	360	400	360
R2	3Ø10	2 Ø10 @110 mm	2 Ø12	40°	Ø10 @ 90 mm	―	360	400	360

Where: α: inclination of the diagonal bars, and ${f}_{yl}$; ${f}_{yd}$; ${f}_{yst}$ are the yield strength of the longitudinal; diagonal; and stirrups, respectively.

### Loading historical

**Fig. 24:**
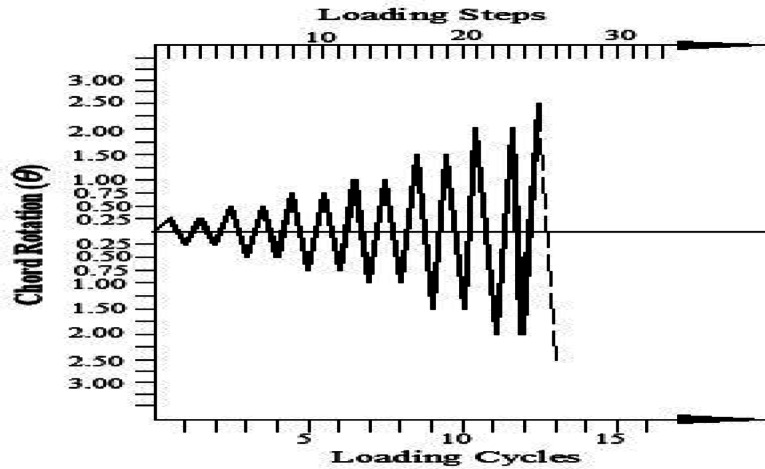
Historical cyclic load.

A cyclic loading protocol was applied to simulate the severe inelastic demand and potential collapse mechanism of coupling beams within a coupled shear wall system during a seismic event. The analysis employed displacement control to facilitate post-yield characterization. The loading history, defined in terms of chord rotation θ = Δ/ln (where Δ is lateral displacement and ln is the beam’s clear span), is illustrated in Fig. 24. The protocol consisted of two complete cycles at each progressively increasing amplitude. The rotation amplitude increased in increments equivalent to 0.25% chord rotation until reaching 1.0%, after which the increment was doubled to 0.50% to efficiently drive the models to failure. Failure was defined as the point where the lateral load capacity degraded to 80% of the peak recorded strength; all subsequent data were excluded from the analysis. It is recognized that this symmetrical, incremental protocol differs from the random load nature of actual earthquake ground motions, but it serves as a standardized and severe test for comparative performance evaluation.

## Results and Discussions

The hysteretic behavior (load versus rotation curves) and key performance characteristics—including crack propagation, failure modes, shear capacity, stiffness degradation, and energy dissipation—were extracted from the finite element models. The results were then analyzed to evaluate the influence of reinforcement schemes and the presence of openings on the structural behavior of the coupling beams.

### Crack initiation, propagation, and failure modes

An analysis of the failure mechanisms is provided based on the analysis data, focusing on the contrasting patterns of crack propagation and the subsequent failure modes.

### Effect of reinforcement details

**Fig. 25:**
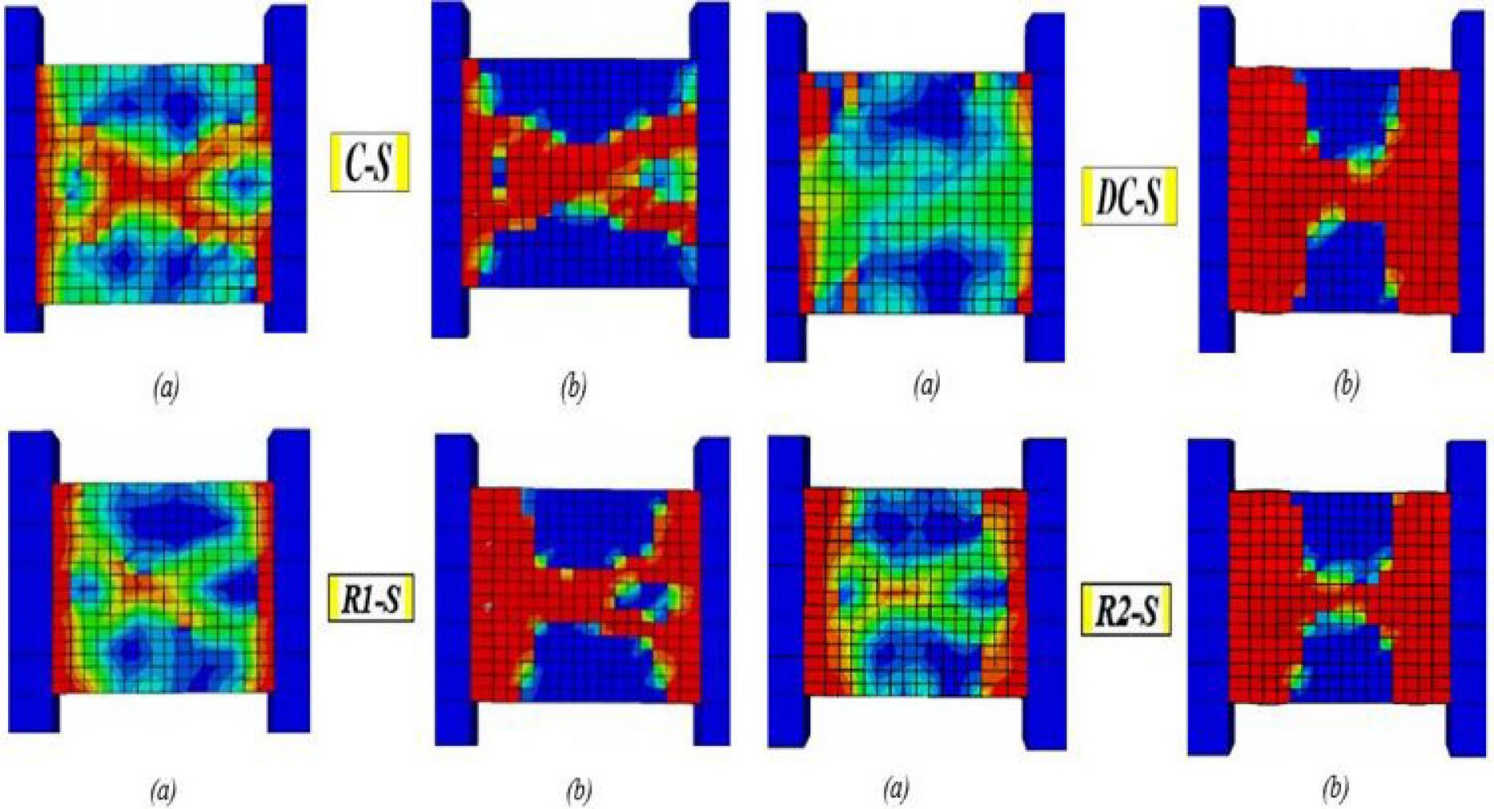
Damage patterns in solid specimens: (**a**) crack pattern and (**b**) concrete crushing.

Based on the data illustrated in Fig. 25, the observed crack propagation and final failure modes for the solid models are analyzed and discussed.

The specimen C-S exhibited a classic brittle shear failure. Cracks initiated at the beam corners, quickly followed by two major diagonal shear cracks at a 45° angle ((a)). As load increased, these cracks propagated severely, leading to sudden failure. The beam ultimately failed in a diagonal shear failure accompanied by significant shear-sliding.

Beam DC-S, featuring diagonal reinforcement, effectively controlled crack propagation by preventing diagonal cracks from reaching the beam ends; cracks became nearly horizontal at mid-span. ((a)). Failure involved diagonal concrete crushing ((b)) along the compressive strut with severe damage at the beam ends. After significant inelastic deformation and energy dissipation, the specimen ultimately failed in a ductile shear mode accompanied by reinforcement yielding.

Beam RI-S initially delayed cracking but lost effectiveness at higher loads, developing a crack pattern similar to conventional specimen C-S ((a)). It ultimately failed through combined severe sliding and diagonal shear ((b)), though at higher inelastic deformation and energy dissipation than C-S.

Beam R2-S exhibited a similar crack pattern of specimen R1-S but the cracks followed a different angle ((a)). Its ultimate failure involves concrete crushing along the compressive strut accompanied with severe damage ((b)) similar to specimen DC-S. The failure is less brittle than C-S and R1-S but not as ductile as DC-S.

### Effect of edge openings

**Fig. 26:**
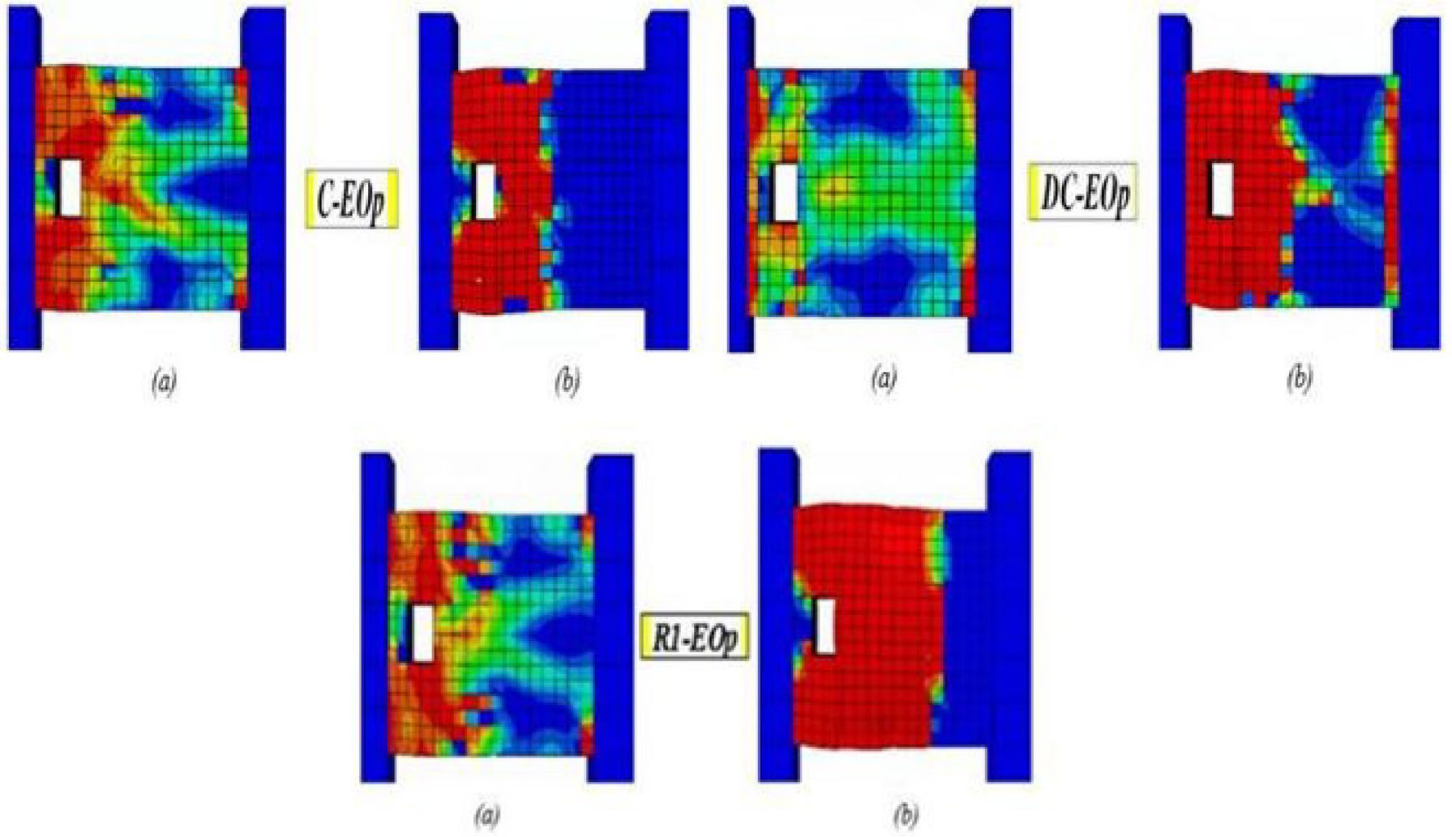
Damage patterns in specimens with end openings: (**a**) crack pattern and (**b**) concrete crushing.

As depicted in Fig. 26, providing specimens with end-span opening (at 37.5 mm from the beam edge) changed the structural behavior and failure mechanisms of all coupling beam types, compared to their corresponding solid models. The openings create severe stress concentrations at their corners, causing localized cracking around the openings.

Beam C-EOp exhibited the most severe and uncontrolled cracking. Cracks propagated directly from the corners of the openings and quickly linked to form a critical failure plane that bypassed the weakened sections ((a)). The beam ultimately experienced a brittle shear failure, resulting in complete damage localized at the end-span region where the opening was placed ((b)).

The diagonal bars (specimen DC-EOp) partially mitigated the adverse effect of the opening, producing a more distributed crack pattern than in specimen C-EOp. Although cracks still initiated at the opening corners, their propagation was partially controlled and followed paths influenced by the diagonal reinforcement ((a)). However, under high load levels, this detailing could not prevent concrete crushing at the critical segment—the end-span region adjacent to the opening ((b)). Consequently, the beam failed in a manner similar to C-EOp, but with significantly enhanced ductility, allowing for greater shear deformation and energy dissipation.

The opening severely diminished the effectiveness of the rhombic reinforcement (beam R1-EOp). The bars failed to control cracks originating at the opening, leading to severe damage in the adjacent end-span as the applied load increased. This resulted in a brittle shear failure, demonstrating performance comparable to the C-EOp specimen.

### Effect of mid-span openings

**Fig. 27:**
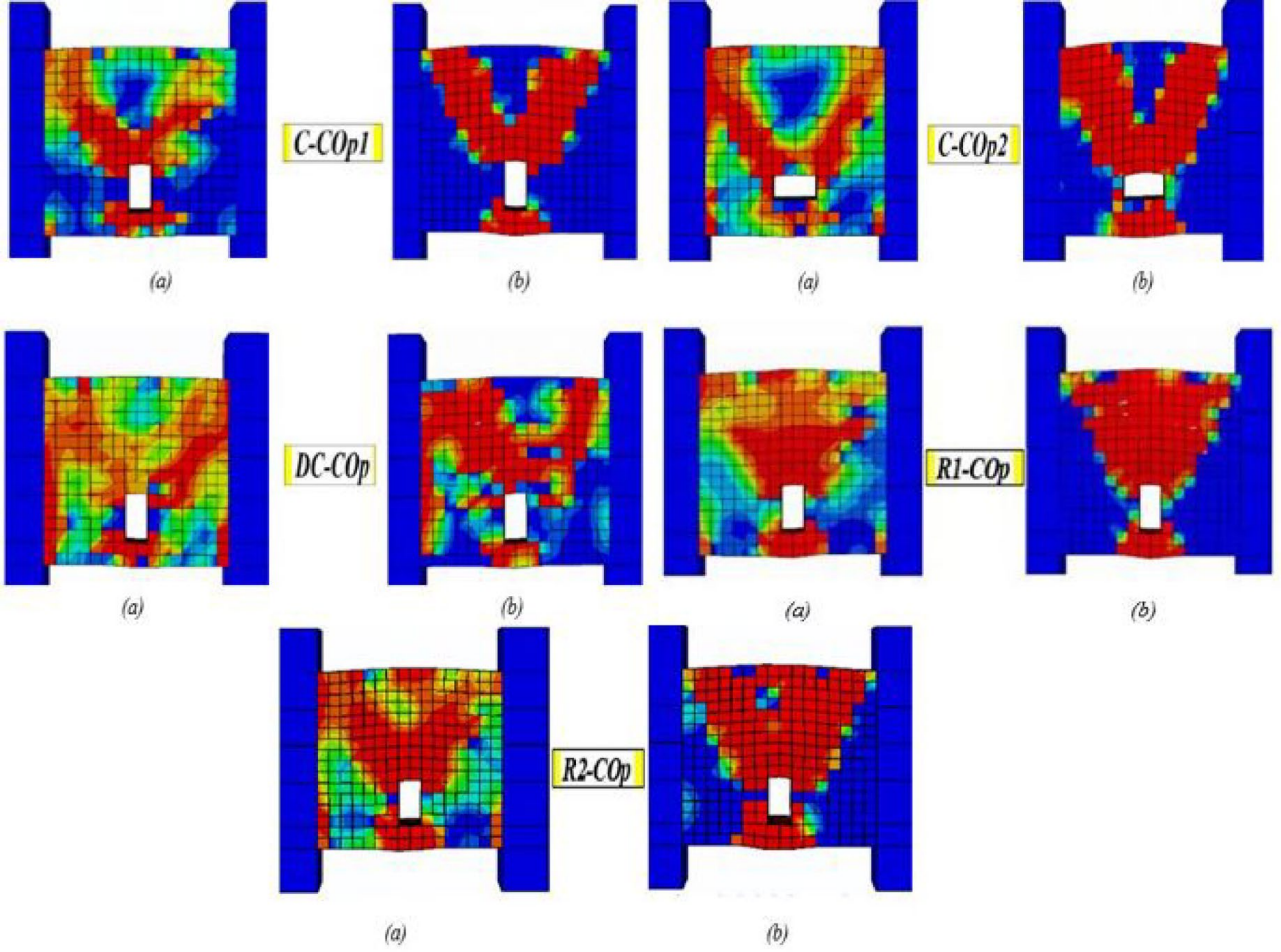
Damage patterns in specimens with mid-span openings: (**a**) crack pattern and (**b**) concrete crushing.

The introduction of a mid-span opening interrupts the primary diagonal compressive strut that forms between beam ends, fundamentally altering the shear-resisting mechanism. Consequently, crack patterns shift from a single major diagonal crack to two dominant diagonal cracks initiating at the opening corners and propagating toward the beam ends. Across all reinforcement details, the presence of an opening significantly increased brittleness compared to solid beams. Figure 27a, b shows the crack propagation and damage for the beams with mid-span openings.

Beam C-Cop1 showed the least controlled and most severe cracking. Two dominant diagonal cracks initiated from the opening corners and propagated towards the beam ends ((a)). This creates a "V-shaped" crack pattern centered on the opening. The vertical stirrups are ineffective in bridging the strong discontinuity caused by the opening. Consequently, rapid crack widening led to a sudden and brittle diagonal shear failure ((b)).

Specimen C-Cop2 exhibited a crack propagation and failure mode similar to those of specimen C-C0p1, but with improved ductility—demonstrated by greater deformation capacity and energy dissipation—prior to the crushing of the confined concrete. Diagonal confinement detail (beam DC-Cop) mitigated the propagation and widening of both the diagonal cracks and those around the opening, thereby preventing a brittle failure. The failure is more gradual, allowed for substantial shear deformation and energy dissipation as the diagonal bars yield. It is markedly superior to other details. Both rhombic details, beams R1-Cop and R2-Cop, showed similar crack propagation and failure, though R2-C0p failed with slightly higher ductility. Cracks initiated the opening corners and propagated towards the beam ends, widening severely under load and leading to brittle diagonal shear failure. The rhombic schemes were significantly weakened by the central opening compared to its corresponding solid beams.

### Shear-rotation responses

**Fig. 28:**
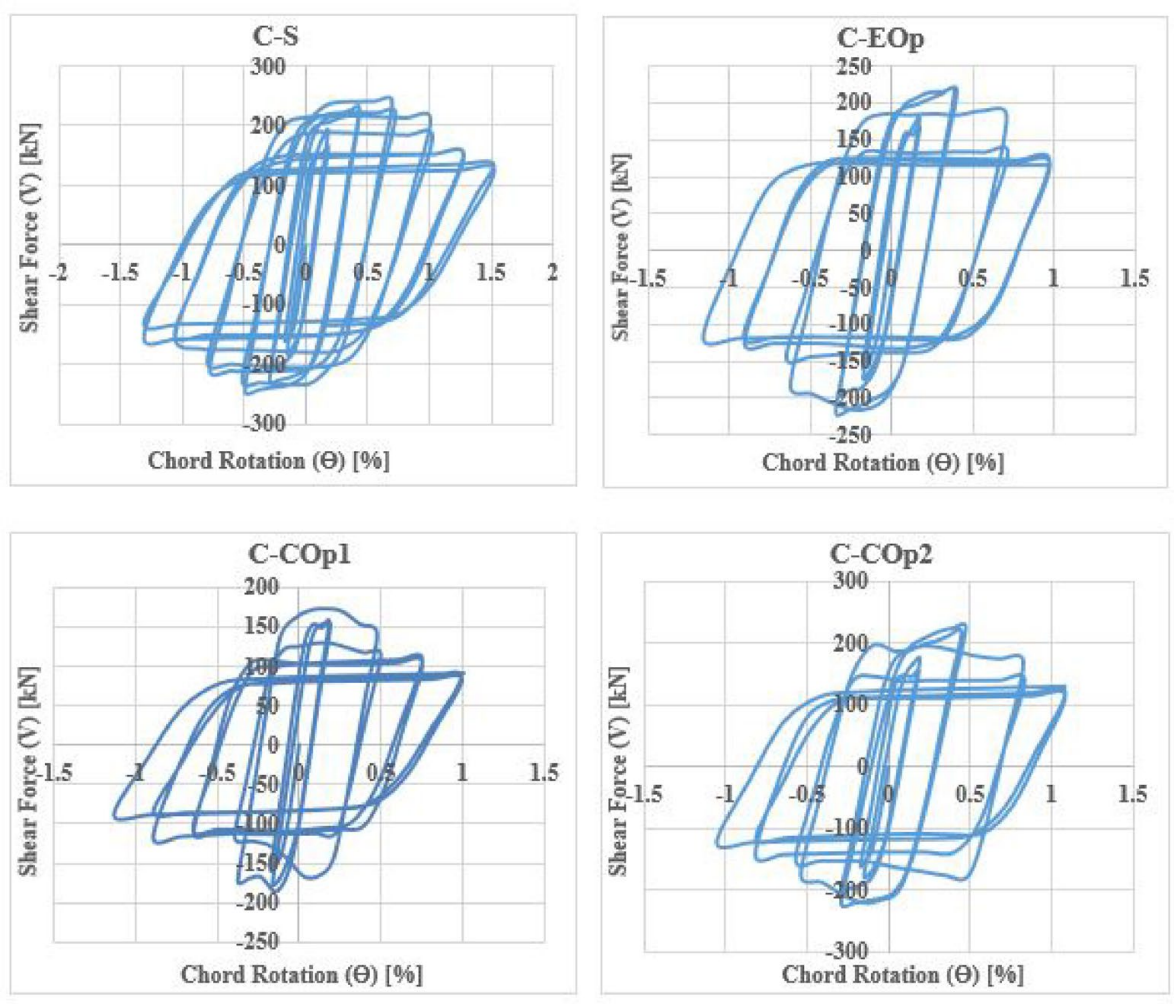
Shear versus chord rotation hysteretic responses for Group C specimens.

**Fig. 29:**
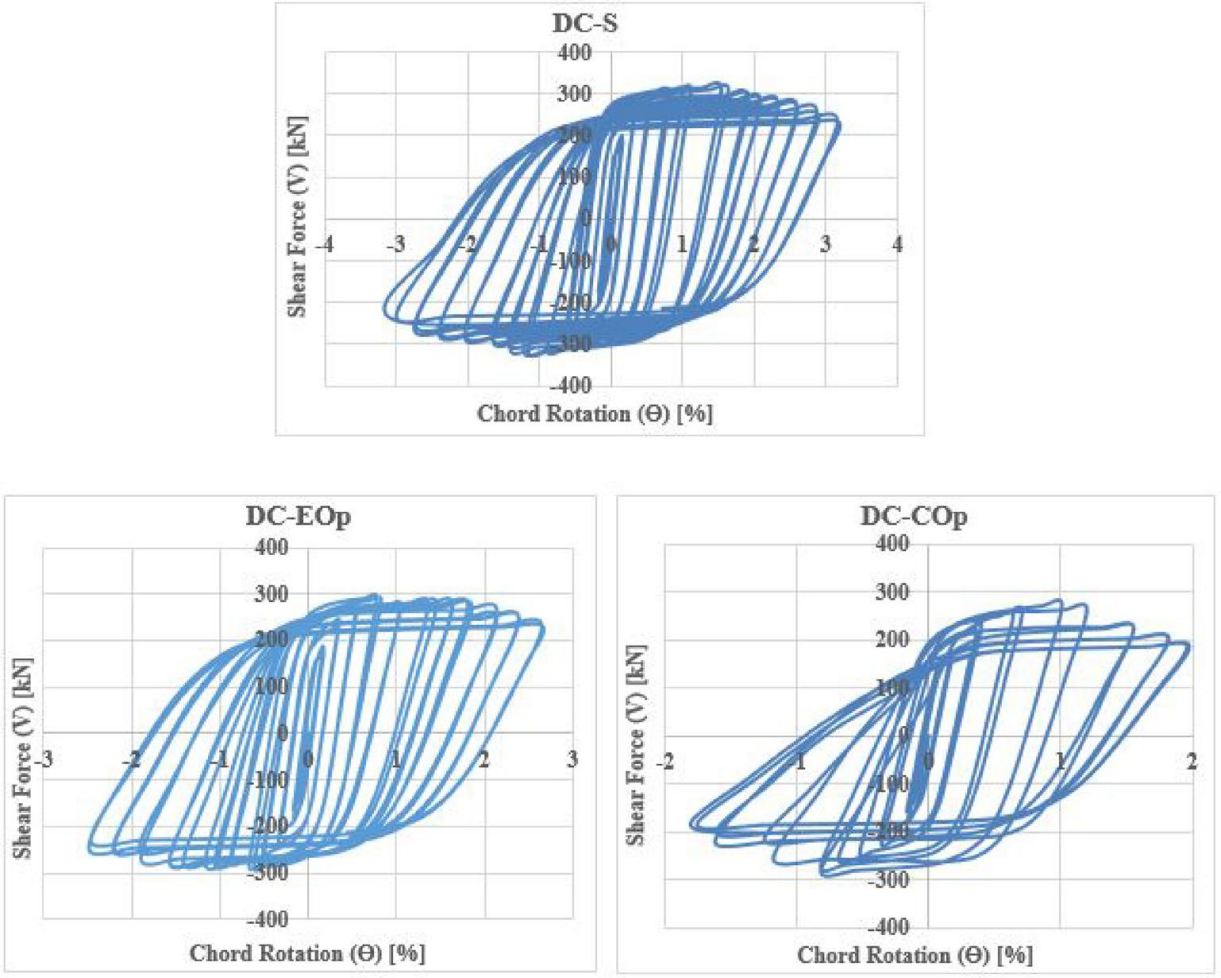
Shear versus chord rotation hysteretic responses for Group DC specimens.

**Fig. 30:**
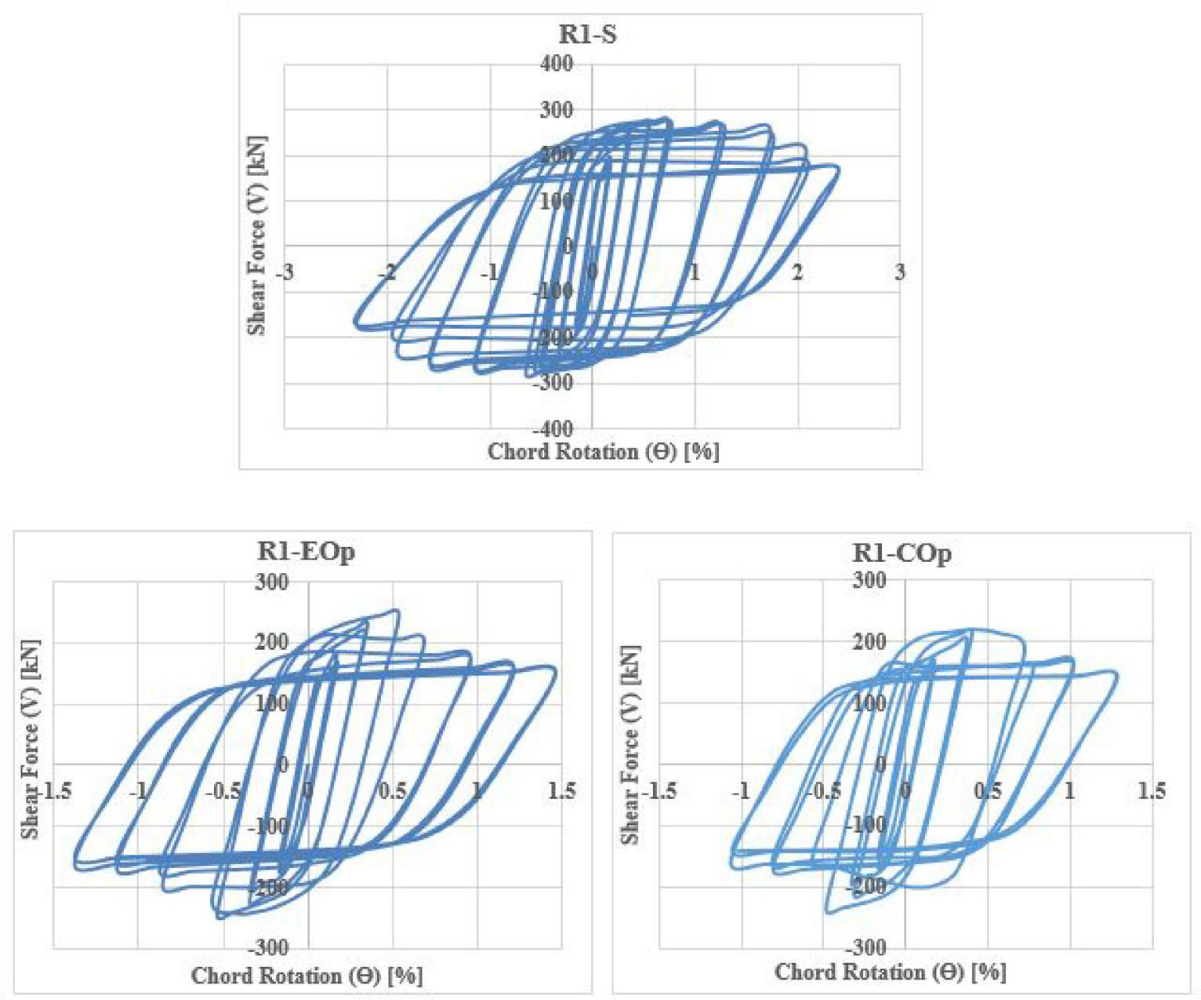
Shear versus chord rotation hysteretic responses for Group R1 specimens.

**Fig. 31:**
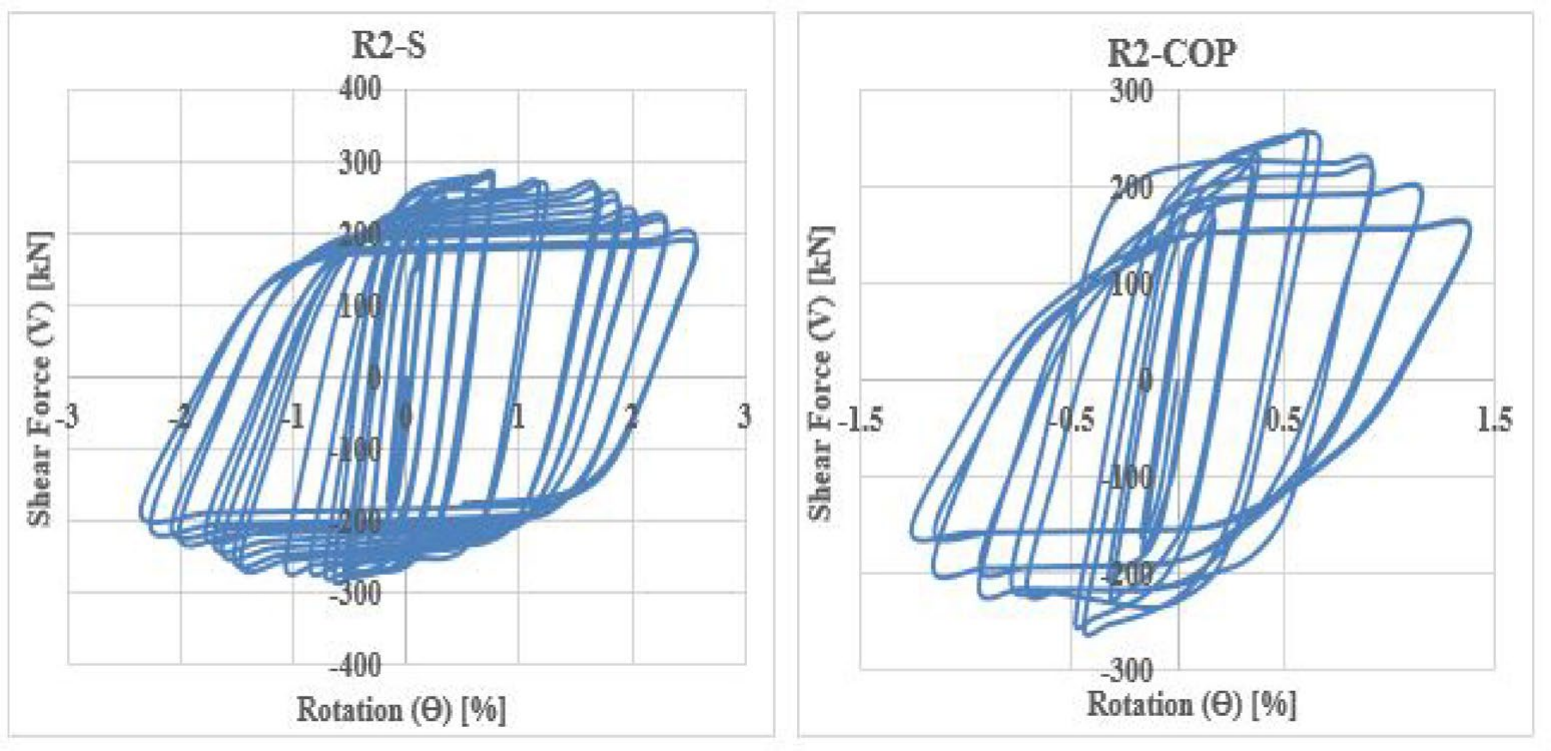
Shear versus chord rotation hysteretic responses for Group R2 specimens.

The shear force versus rotation (V-θ) relationship reveals key characteristics of the seismic behavior of coupling beams (CBs), including shear capacity, ductility, stiffness degradation, and energy dissipation. The cyclic responses for all specimens are shown in Figs. 28, 29, 30 and 31.

**Fig. 32:**
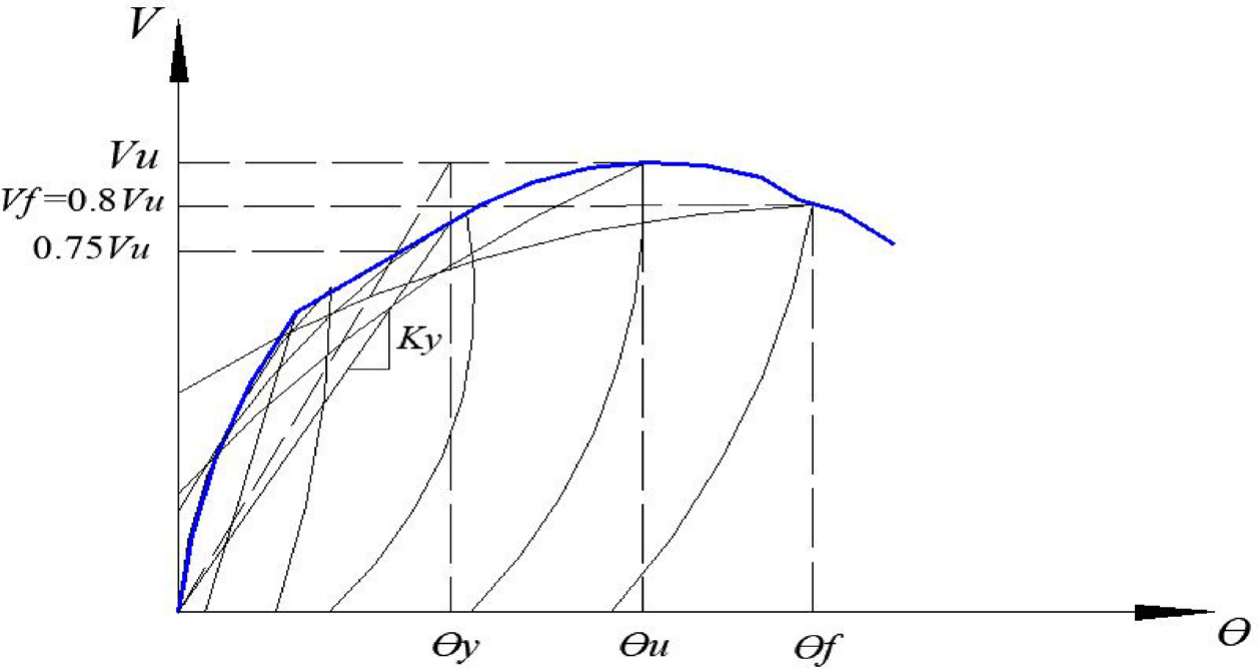
Definition of key performance parameters from the backbone envelope curve.

Key performance parameters were extracted from the envelope curves, as illustrated in Fig. 32 and summarized in Table 4. These parameters are:

**Table 4:** Key parameters from the hysteretic response envelope.

Specimen	Vy (kN)	Vu (kN)	θy (%)	θu (%)	θf (%)	µ	Ky (kN/rad)
C-S	221.6	242.6	0. 25	0.7	1.02	4.08	88,640
C-EOp	187.3	217.9	0.19	0.41	0.71	3.74	98,579
C-COp1	151.6	172.7	0.13	0.2	0.42	3.23	116,615
C-COp2	201.5	227.4	0.22	0.47	0.83	3.77	91,591
DC-S	300.4	318.8	0.56	1.55	3.2	5.71	53,643
DC-EOp	283.4	301.2	0.52	0.84	2.7	5.19	54,500
DC-COp	256.8	287.8	0.46	1.01	1.64	3.57	55,826
R1-S	279.1	290.3	0.43	0.81	2.12	4.93	64,907
R1-EOp	221.6	253.2	0.32	0.53	0.95	2.97	69,250
R1-COp	205.4	219.9	0.25	0.4	0.73	2.92	82,160
R2-S	270.4	280.1	0.42	0.81	2.31	5.5	64,381
R2-COp	218.6	247.3	0.30	0.62	1.15	3.83	82,333

- **V**y: Yield shear force.
- **V**u: Ultimate shear capacity.
- **V**f: Failure load (defined as a 20% strength drop from Vu).
- θy: Yield rotation.
- θu: Ultimate chord rotation.
- θf: Chord rotation at failure.
- **Ky**: Stiffness at the yield point.

The yield point was determined according to^29^ as the intersection of the line representing the ultimate load and a secant line from the origin through the point at 0.75Vu.

The results clarified that, solid beams with diagonal and rhombic details (specimens DC-S, R1-S, and R2-S) exhibited a stable hysteretic behavior compared to that with conventional detail (C-S). Specimen DC-S exhibited the highest performance with gradual capacity degradation and highest number of cycles up to failure compared to the other details (specimens C-S, R1-S, and R2-S). In all cases, the presence of opening has showed an effect on the hysteretic behavior of coupling beams: decreased the ductility and exhibited highly rapid failure, thus lower number of cycles up to failure, especially when the opening is located at the mid-span of the beam. In case of conventional specimens, producing the opening near the end of the beam (C-EOp) decreased the number of cycles up to failure from 8 to 6 cycles compared to the corresponding solid beam (C-S). Specimen with mid-span opening (C-COp1) is highly affected by the opening and exhibited highly brittle failure (failed after 4 cycles only). Changing the opening dimensions in the center-span of the beam, specimen (C-COp2), mitigated the opening effect and improved the hysteretic behavior compared to specimen C-COp1. Specimen (DC-EOp) exhibited a stable hysteretic behavior during loading cycles and succeeded in undergo high large rotation up to failure. Shifting the opening to the mid-span of the beam (specimen DC-COp) had a significant effect on the hysteretic behavior and maximum chord rotation, and decreased the number of cycles. Producing the opening in rhombic details had a significant effect on the hysteretic behavior else the opening located near the end-span or at the mid-span of the coupling beam.

### Shear capacity

The introduction of an opening in a coupling beam reduces its effective cross-sectional depth, which directly diminishes its capacity to resist shear and flexural stresses. Consequently, the presence of an opening significantly affects the ultimate shear capacity.

**Fig. 33:**
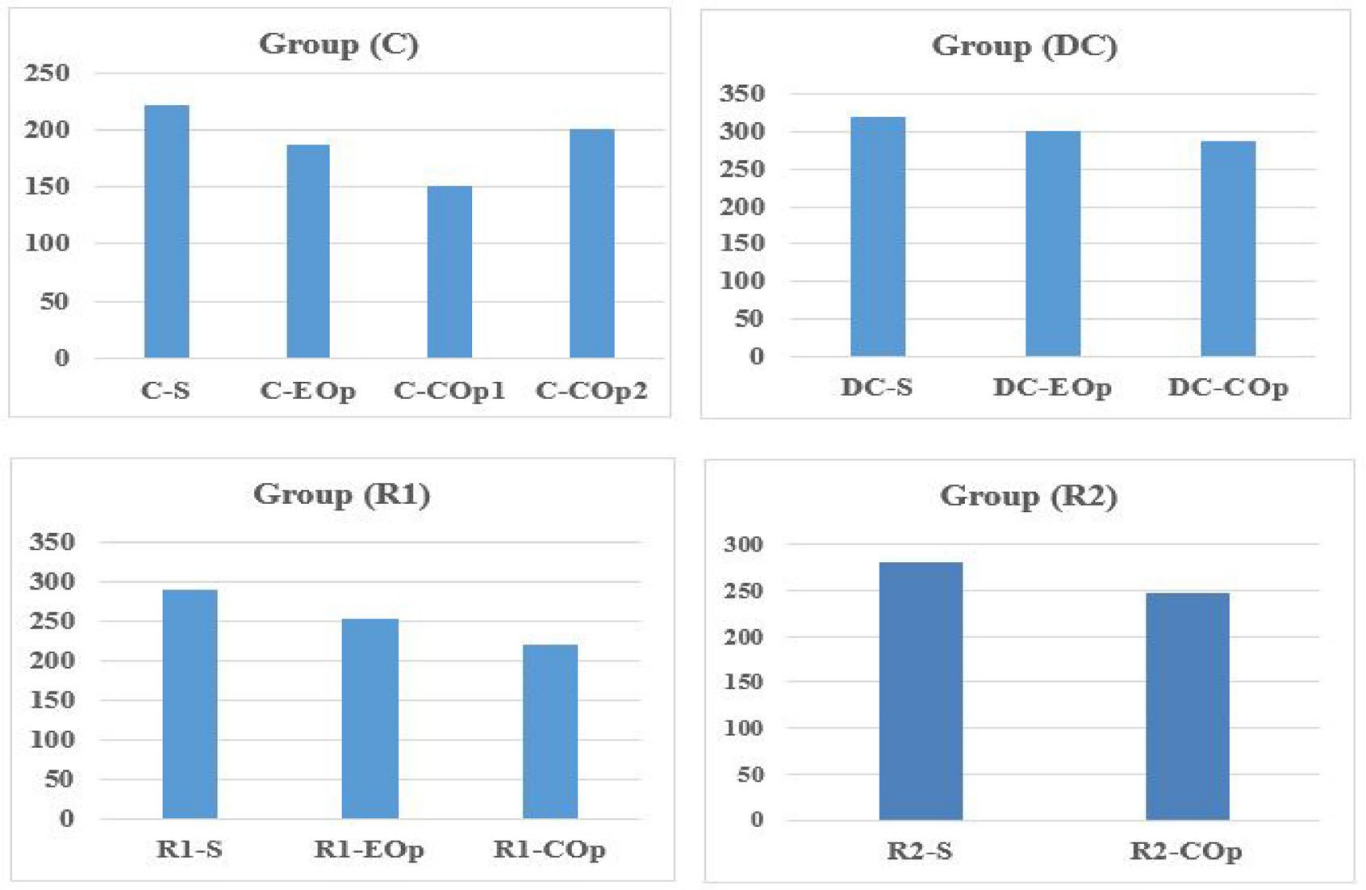
Comparison of ultimate shear capacity (Vu) (kN) between solid and perforated coupling beams.

Table 4 and Fig. 33 compare the shear capacities of solid coupling beams (CBs) with different reinforcement details to their counterparts with openings. The comparison of shear capacities reveals that the introduction of openings consistently reduces strength across all reinforcement types, but the magnitude of this detrimental effect is highly dependent on the detailing strategy and the opening location.

For edge openings, the reduction in ultimate shear capacity (Vu) relative to solid beams was 10.2% for conventional detailing (C), a minimal 5.5% for diagonal confinement (DC), and 12.8% for rhombic configuration 1 (R1). These results confirm that diagonal confinement is the most effective strategy, losing under 6% in strength—likely because diagonal bars effectively bypassing the edge discontinuity and forming a continuous corner-to-corner tie.

The effect was more significant for central openings (COp), with reductions of 31.6% (C), 14.5% (DC), 26.4% (R1), and 19.2% (R2). Thus, the effectiveness of reinforcement detailing in mitigating the weakening effect of the mid-span opening clearly ranked: diagonal confinement (DC) is superior, exhibiting the smallest reduction in shear capacity. It is followed by Rhombic Detail 2 (R2), which shows greater resilience than Rhombic Detail 1 (R1). Conventional reinforcement (C) proves the least effective, consistently exhibiting the most severe degradation in strength.

The data clearly demonstrates that the impact of an opening on shear capacity is profoundly influenced by its location, with edge openings causing a markedly less severe reduction than central openings across all reinforcement types. This is because an edge opening primarily disrupts the shear transfer and anchorage zone at the beam-wall junction, whereas a central opening interrupts the primary diagonal compression strut that forms across the entire beam span under shear, a more critical load path.

A notable exception was specimen C-COp2, where inverting the opening dimensions changing from (lo/l = 0.125 and ho/t = 0.25) to (lo/l = 0.25 and ho/t = 0.125) drastically reduced the detrimental effect, lowering the capacity reduction from 31.6% to only 9.1%. The significant enhancement in coupling beam capacity observed upon altering the dimensions of the opening is fundamentally attributed to moving the opening away from the critical diagonal load path, thereby preserving its structural integrity.

### Ultimate chord rotation and ductility

Coupling beams must be designed to undergo large inelastic rotations and exhibit ductile behavior. Beams with low ultimate chord rotation and ductility are unsuitable for coupled shear wall systems, as they cannot perform their intended function as structural fuses.

Ductility is quantified by the ductility index (µ), defined in reference^23^ as the ratio of the maximum chord rotation at failure to the yield chord rotation:

6 $$\mu = \, \theta {\text{f }}/\theta {\mathrm{y}}$$

The ductility parameters—yield rotation (θy), maximum rotation at failure (θf), and ductility index (µ)—for all beams are compared and discussed in this section.

According to the results in Table 4, the solid beam with diagonal confinement (DC-S) exhibited superior ductility and ultimate chord rotation compared to the conventional (C-S) and rhombic (R1-S, R2-S) specimens. Specimen DC-S achieved a maximum chord rotation (θf) of 3.20%, which is approximately 3.1, 1.5, and 1.4 times greater than that of specimens C-S (1.02%), R1-S (2.12%), and R2-S (2.31%), respectively. Similarly, the ductility index (µ = 5.71) for DC-S was 28.5% higher than C-S (µ = 4.08), 13.7% higher than R1-S (µ = 4.93), and 3.7% higher than R2-S (µ = 5.50). Diagonal detailing is superior because it directly targets the failure mode (diagonal tension), creates a more efficient and ductile load path, distributes inelastic deformation more evenly, and better maintains concrete integrity under large deformations.

### Effect of edge openings

The presence of an edge opening significantly reduced both the maximum chord rotation and ductility. The conventional specimen (C-EOp) experienced significant reductions of 30.4% in θf and 8.3% in µ compared to its solid counterpart (C-S), indicating a substantial loss in its ability to rotate and dissipate energy. Notably, the rhombic-detailed beam (R1-EOp) suffered the most severe weakness, with drastic reductions of 55.2% in θf and 39.8% in µ, suggesting that its reinforcement scheme is particularly vulnerable to the disruption in force transfer and anchorage at the beam end caused by the edge opening. In contrast, the diagonally confined specimen (DC-EOp) demonstrated superior performance. It maintained a much higher proportion of its inherent ductility, with reductions of only 15.6% in θf and 9.1% in µ relative to the solid diagonally confined beam (DC-S). This exceptional performance is achieved through diagonal reinforcement, which creates a continuous load path that bypasses the edge discontinuity. This mechanism preserves rotational capacity and ensures stable post-yield behavior, demonstrating the efficacy of this detailing strategy.

### Effect of mid-span openings

The positioning of the opening at the beam’s mid-span led to a severe degradation in ductility and deformation capacity across all reinforcement types. This effect is most pronounced for beams with conventional and rhombic detailing. For the conventional beam (C-COp1), a central opening resulted in drastic reductions of 58.8% in maximum chord rotation (θf) and 20.8% in ductility index (µ) compared to its solid counterpart. Notably, simply inverting the opening geometry for specimen C-COp2 significantly mitigated this effect, reducing the loss in θf to only 18.6%, which highlights the profound sensitivity of performance to the opening’s specific dimensions and orientation within the critical shear path.

Similarly, central openings critically weakened the rhombic-detailed beams. R1-COp experienced the most severe weakness of all tested specimens, with reductions of 65.6% in θf and 40.8% in µ. The alternative R2-COp configuration fared better but still suffered substantial losses of 50.2% in θf and 30.4% in µ, indicating that while the rhombic detail offers an improvement over conventional layout, it remains vulnerable to a central discontinuity.

Even the superior diagonally confined beam (DC-COp) was not invulnerable, showing significant reductions of 48.8% in θf and 37.5% in µ. Despite, the absolute deformation capacity retained by DC-COp (θf = 1.64%) remained comparable to or even exceeded that of the solid beams with other detailing schemes, underscoring the fundamental resilience of the diagonal load path. That confirms while a central opening is detrimental to all systems, diagonal confinement provides the most robust foundational detailing.

### Stiffness degradation

Analysis of stiffness degradation is used to quantify damage accumulation in reinforced concrete members. The degradation was evaluated by tracking the secant stiffness (K) at each load cycle’s amplitude against the chord rotation (θ).

**Fig. 34:**
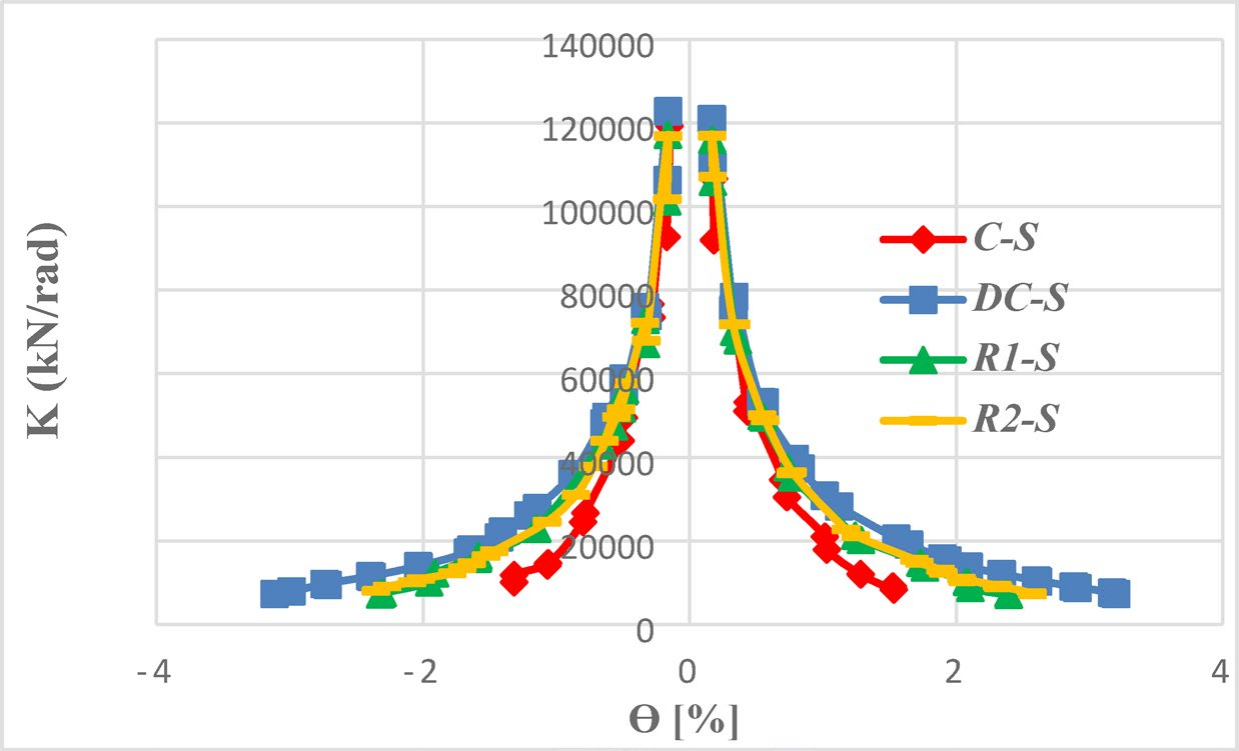
Stiffness degradation of solid beams.

Figure 34 compares the stiffness degradation of solid specimens with different reinforcement layouts. While all solid specimens began with similar initial stiffness and degraded at a comparable rate down to approximately two-thirds of their initial value, their behavior diverged significantly thereafter. The diagonally confined beam (DC-S) demonstrates the most stable and ductile performance and showing a more gradual decline until the end of loading. In contrast, the conventional beam (C-S) exhibited rapid and brittle stiffness loss as the rotation increased. The two rhombic details (R1-S and R2-S) exhibited nearly identical degradation trends, with slightly lower stiffness than the diagonally confined beam, indicating improved stiffness retention over conventional reinforcement. This confirms that while rhombic layouts enhance post-peak performance, diagonal confinement remains the most effective strategy for maintaining superior stiffness and stable, ductile cyclic response.

**Fig. 35:**
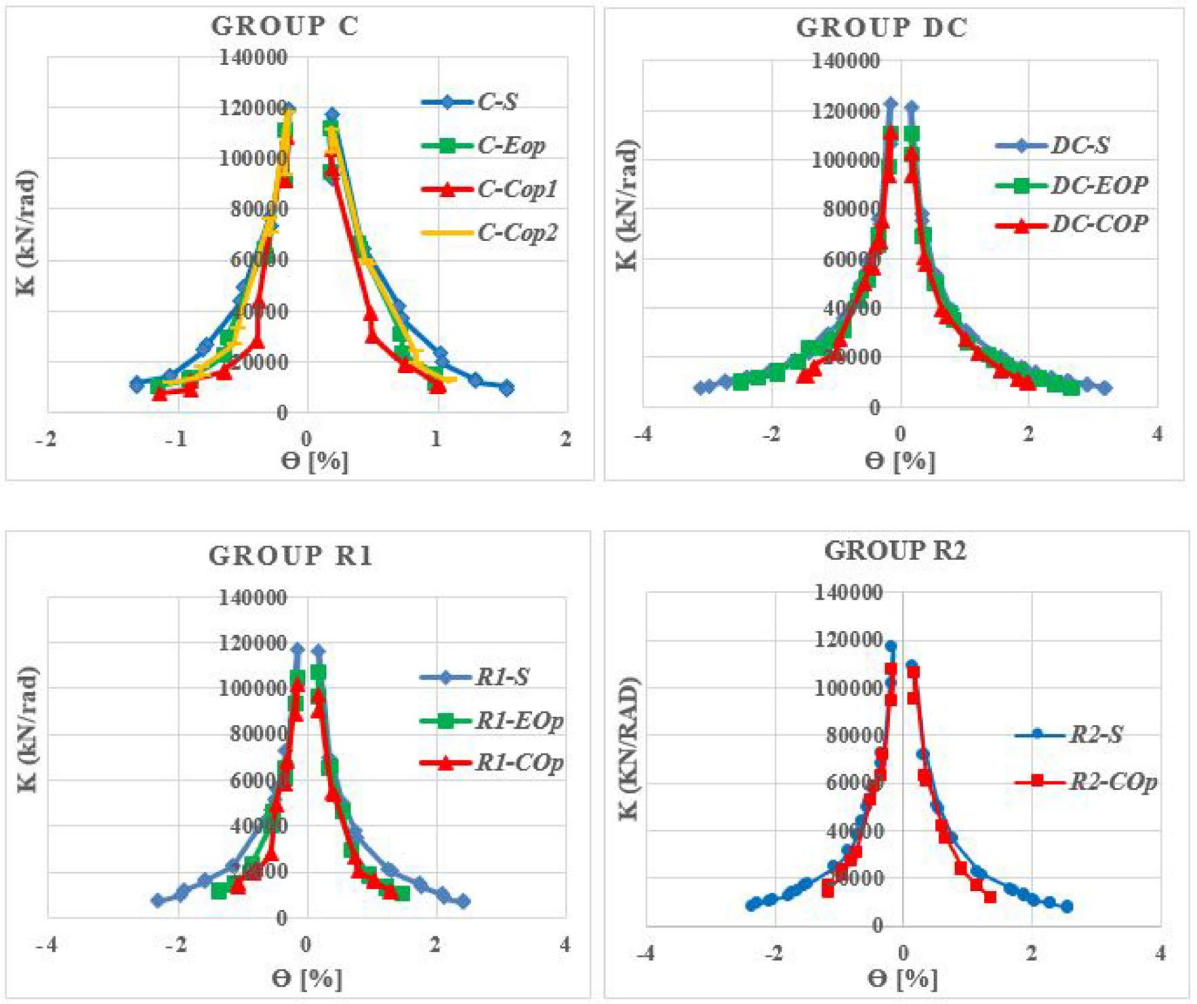
Stiffness degradation of the analyzed beams.

As illustrated in Fig. 35, the presence of the opening accelerated damage progression across all reinforcement types. The most severe stiffness loss was observed in beams containing mid-span openings.

The beam (C-EOp), maintained a similar degradation rate to it solid counterparts until the stiffness was halved. Beyond this point, damage localized around the opening led to an accelerated deterioration in stiffness as loading increased. Shifting the opening to the mid-span (C-COp1) produced a significantly more detrimental response, characterized by a sharp and immediate drop in stiffness at very low drift levels. Inverting the opening geometry in specimen C-COp2 effectively mitigated this detrimental effect, achieving a stiffness retention that closely approached that of the end-opened beam (C-EOp). These findings underscoring the important influence of opening shape—not just location—play a decisive role in the stiffness performance of conventionally reinforced coupling beams.

The diagonally confined detail (DC) was far less affected by openings. The beam (DC-EOp) exhibited exceptional performance; its stiffness degradation curve was nearly identical to that of the solid reference beam (DC-S) throughout the entire loading history. This indicates that the diagonal reinforcement successfully channeled loads around the end opening without a significant loss of structural integrity. The specimen (DC-COp) also maintained stiffness behavior closely aligned with the solid specimen until approximately 50% of the initial stiffness was lost. After that, the presence of the opening became more influential, leading to an increased rate of degradation.

The two beams (R1-EOp) and (R1-COp) displayed nearly identical degradation paths, except that the beam with end opening has initial stiffness higher than the other one. The presence of the opening had a relatively minor effect on stiffness degradation in the initial loading stages. However, at higher rotation levels, a significant acceleration in stiffness loss occurred as the applied load increased, indicating a delayed but critical weakening of the internal rhombic truss mechanism as damage accumulated around the opening.

The rhombic detail R2 offered a moderate improvement in performance for beams with a mid-span opening. However, this enhancement was insufficient to prevent accelerated stiffness degradation at higher rotational drifts. A comparative analysis reveals that the stiffness degradation curve of specimen (R2-COp) matched that of the solid R2 beam (R2-S) until approximately two-third of the initial stiffness was lost. Beyond this point, the presence of the opening in R2-COp became dominant, triggering a pronounced decline in stiffness.

### Energy dissipation

Energy dissipation capacity is a key parameter for evaluating the seismic performance of coupling beams. It is calculated as the cumulative area enclosed by the shear-rotation hysteresis loops.

**Fig. 36:**
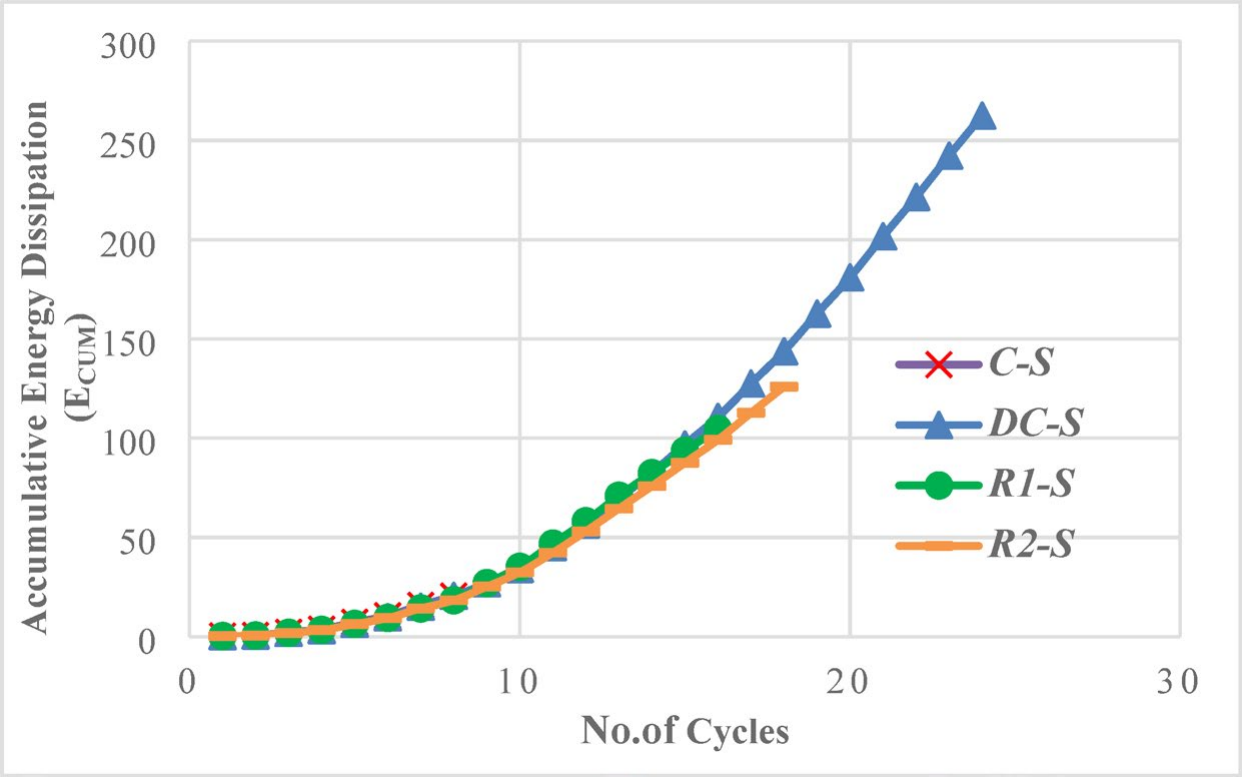
Comparison of cumulative energy dissipation for solid specimens with different reinforcement details.

Figure 36 compares the cumulative energy dissipation (Ecum) for the solid specimens. The results demonstrate that the reinforcement detail has a significant effect on the total absorbed energy. Specimen DC-S exhibited superior performance, sustaining large rotations over a high number of cycles and achieving a total energy dissipation of 262.5 kN·rad. In contrast, specimen C-S displayed brittle failure after only eight cycles, resulting in the lowest total energy dissipation of 21.9 kN·rad. The rhombic specimens (R1-S and R2-S) performed better than the conventional one, achieving total energy dissipations of 104.6 kN·rad and 125.9 kN·rad, respectively. The diagonal detail’s marked improvement is achieved from its reinforcement layout aligning with principal tensile stresses. This promotes uniform inelastic strain distribution, delays critical failure mechanisms, and significantly enhances ductility and energy dissipation.

**Fig. 37:**
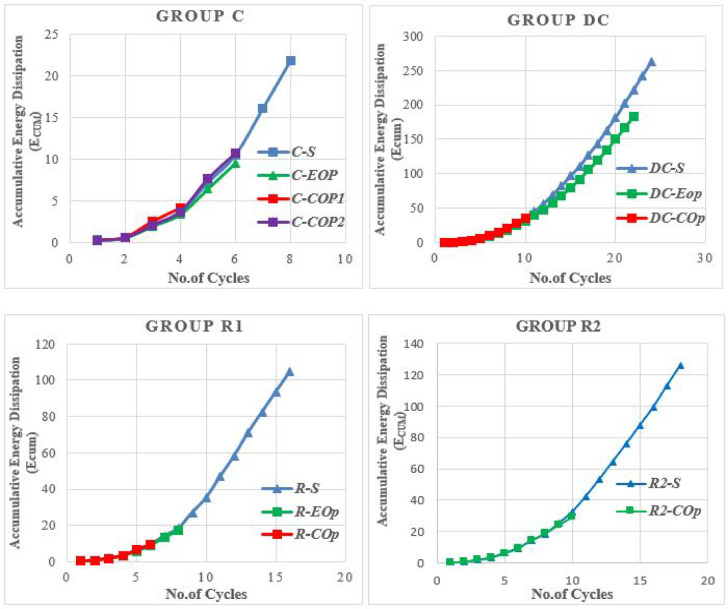
Comparison of cumulative energy dissipation for analyzed specimens.

The cumulative energy dissipation for the specimens with openings was also analyzed and compared to their solid counterparts in Fig. 37. The presence of openings generally reduced ductility, leading to rapid failure, fewer cycles, and lower energy dissipation, especially for mid-span openings.

The conventional scheme proved least effective at mitigating energy dissipation losses caused by openings. In the end-opened specimen (C-EOp), dissipation was only slightly affected at small drifts, but the opening’s impact emerged at higher load levels, ultimately precipitating premature failure. The total dissipated energy of 9.5 kN.rad represented a severe 56.6% reduction compared to the solid beam. Mid-span openings (C-COp1) were even more detrimental, causing rapid failure at a very low number of cycles with energy dissipation of only 4.2 kN.rad. However, modifying the opening geometry by inverting its aspect ratio (C-COp2) improved performance substantially, increasing energy dissipation to 10.7 kN.rad and producing more stable hysteretic behavior. This demonstrates that while conventional detailing is highly vulnerable to openings, geometric optimization of the opening itself can offer significant improvement.

The diagonally confined detail demonstrated superior performance, sustaining large rotations over many cycles with a total energy dissipation of 262.5 kN·rad. Furthermore, it provided the most effective in preserving energy dissipation capacity in the presence of openings. With the end opening (DC-EOp), the beam retained considerable performance, dissipating 182.9 kN.rad—only a 30.3% reduction from the solid specimen. It maintaining stable hysteresis up to total energy dissipation about 50 kN.m, then the capacity of beam to dissipate energy gradually decreased. Even with a mid-span opening (DC-COp), which failed in less ductile manner and caused a more significant 86.6% reduction, the beam still dissipated 35.2 kN.rad, which was the highest among all perforated specimens.

The Detail R1 showed limited effectiveness in mitigating the energy dissipation loss caused by openings, performing only marginally better than conventional reinforcement in some cases. The end-opened specimen (R1-EOp) dissipated 17.6 kN.rad, an 83.1% reduction from its solid counterpart—the largest reduction among EOp specimens. Its performance with a mid-span opening (R1-COp) was even poorer, dissipating only 9.5 kN.rad, a 90.9% reduction. This indicates that the R1 detail is highly sensitive to discontinuities, as its mechanism is apparently disrupted by openings regardless of location, resulting in rapid stiffness degradation and low cyclic energy absorption.

The second rhombic detail (R2) provided better resistance to energy dissipation loss for mid-span openings than R1. The mid-span opened beam (R2-COp) dissipated 29.1 kN.rad, a 76.9% reduction from the solid beam. Although this still represents a major decrease, it is notably better than the 90.9% reduction seen in R1-COp. Nevertheless, R2-COp’s energy dissipation remained below that of DC-COp (35.2 kN·rad), suggesting that rhombic2 pattern didn’t surpass the effectiveness of diagonal confinement in maintaining energy absorption in perforated beams.

## Conclusion

This study investigated the cyclic behavior of short reinforced concrete coupling beams (RCCBs) with different reinforcement details—conventional (C), diagonal confinement (DC), and two rhombic configurations (R1 and R2)—and examined the effect of web openings. The primary variables were the reinforcement scheme, opening location, and opening dimensions. The key findings lead to the following practical guidance.

1. Recommendation for reinforcement detailing

The results clearly establish a performance hierarchy, which should directly inform design choices, especially when openings are necessary:

- First Choice (Diagonally Confined—DC): The most robust and resilient detail —demonstrates superior performance. It’s recommended to be the standard detail for short coupling beams, particularly when openings are necessary.
- Acceptable alternative (Rhombic—R1 or R2): It offers a good balance if DC detailing is challenging. However, its performance degrades sharply in the presence of openings, particularly those at the mid-span. Therefore, if service openings are anticipated, the more robust DC detail is mandatory.
- Not recommended: the conventional (C) detail, especially with openings. It showed brittle failure and drastic loss of energy dissipation which makes it seismically vulnerable.

2. Opening location: a critical design parameter

- Edge openings (EOp): less detrimental. Performance reductions, while significant, were managed best by the DC detail with only (5.5% and 15.6%) reduction in the shear capacity and maximum chord rotation, respectively.
- Mid-Span openings (COp): extremely detrimental. Intersect with the primary diagonal compression strut, short-circuiting the load path. Reductions in chord rotation and energy dissipation were catastrophic (often > 50%, up to 90.9%).
- Practical guidance: when an opening is necessary at the mid-span, its geometry must be designed to preserve the integrity of the diagonal compressive strut—the critical shear-resisting load path between the beam ends. While a mid-span opening does not intersect this critical path, it may have a less severe impact on performance than an end opening that does.

3. Proposed dimensional limits for openings

The study on opening dimensions (lo/l and ho/t) provides a basis for quantitative limits.

- Key Finding: A long, low opening (lo/l = 0.25, ho/t = 0.125) was far less damaging than a short, tall one (lo/l = 0.125, ho/t = 0.25), even with the same area. This underscores that opening height is the dominant geometric factor.
- Proposed Limits (for Short Coupling Beams, l/h = 1):
- Maximum Height (ho/t): The results demonstrate that opening height is a critical parameter; a height-to-thickness ratio (hₒ/t) of 0.25 caused severe damage, whereas a ratio of 0.17, as shown in the literature^22^, has a minimal effect. The governing design principle is that the opening must not interrupt the diagonal concrete strut, which is the primary shear-resisting mechanism, particularly for openings near the mid-span.
- Maximum length (lo/l): limit to ≤ 0.25 of the clear span.
- Aspect ratio: prefer longitudinal-oriented openings (length > height).
- Edge distance: the opening’s edge should be no closer than 0.25 h from the beam face, and must outside the diagonal compressive strut path at mid-span.

4. Implications for modeling and analysis

- The validated FEM model is a powerful tool but requires careful implementation. The findings and proposed dimensional limits are derived specifically for short coupling beams (l/h = 1) where shear behavior predominates. Beams with different geometries or where openings exceeding these limits requires further verification.

### Suggested future work

1. Extending the work to coupling beams with larger aspect ratios where flexural behavior may dominate, as well as investigating coupling beams executed using high-strength concrete.
2. Utilizing the conclusions of published experimental and theoretical research to develop standard guidelines and reinforcement detailing that can be directly incorporated into design codes.

## Data Availability

All data generated or analyzed during this study are included in this published article.
